# Engineering Assembloids to Mimic Graft‐Host Skeletal Muscle Interaction

**DOI:** 10.1002/adhm.202404111

**Published:** 2025-05-05

**Authors:** Lucia Rossi, Beatrice Auletta, Luigi Sartore, Marco La Placa, Giada Cecconi, Pietro Chiolerio, Edoardo Maghin, Silvia Angiolillo, Eugenia Carraro, Onelia Gagliano, Cecilia Laterza, Nicola Elvassore, Martina Piccoli, Anna Urciuolo

**Affiliations:** ^1^ Department of Molecular Medicine University of Padova Via G. Colombo 3 Padova 35131 Italy; ^2^ Neuromuscular Engineering lab Istituto di Ricerca Pediatrica Città della Speranza Corso Stati Uniti 4/F Padova 35127 Italy; ^3^ Department of Industrial Engineering University of Padova Via Gradenigo 6/a Padova 35131 Italy; ^4^ Tissue Engineering lab Istituto di Ricerca Pediatrica Città della Speranza Corso Stati Uniti 4/F Padova 35127 Italy; ^5^ Department of Chemical and Pharmaceutical Sciences University of Trieste Trieste 34127 Italy; ^6^ Veneto Institute of Molecular Medicine Via Orus 2 Padova 35131 Italy; ^7^ Department of Biomedical Sciences University of Padova via Ugo Bassi 58/B Padova 35131 Italy

**Keywords:** assembloids, muscle regeneration, muscle stem cell, neuromuscular organoids, organoids, skeletal muscle, tissue engineering

## Abstract

Skeletal muscle (SkM) tissue engineering aims to generate in vitro 3D products that can be implanted in patients to replace or repair damaged muscles. Having a humanized in vitro model able to mimic the interaction between the innervated recipient and the engineered SkMs at a functional level would greatly help in the evaluation of the graft potential. Here, a 3D in vitro model is developed that allows to investigation of the function, stability, and adaptability of the human neuromuscular (NM) system in response to an engineered SkM construct. To achieve this, decellularized SkMs (dSkM)‐based constructs are used as engineered SkM and human neuromuscular organoids (NMOs) as the recipient‐like NM system to create graft‐host SkM assembloids. We observed the migration of myogenic cells and invasion of neural axons from the NMO to the engineered SkM construct in the assembloids, with the generation of functional neuromuscular junctions (NMJs). Finally, assembloids are able to regenerate following acute damage, with SkM regeneration and functional recovery. Despite being limited by the absence of immunocompetent cells and vasculature, the data showed that the assembloid represents a useful tool to evaluate in vitro the response of the human innervated SkM to a potential tissue‐engineered SkM graft.

## Graphical abstract

This study develops a graft‐host skeletal muscle assembloid model combining neuromuscular organoids with tissue‐engineered constructs. Pre‐seeding decellularized muscles with myogenic cells enhances cell migration and axon invasion from the organoid. The model exhibits regenerative capacity following acute damage, advancing the understanding of human neuromuscular system regeneration and offering a valuable in vitro tool for evaluating potential tissue‐engineered skeletal muscle grafts.

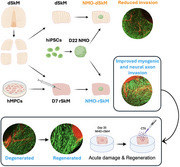

## 1 Introduction

One of the main goals of tissue engineering is to generate in vitro organs and tissues that can be implanted in vivo to replace or repair diseased and damaged ones.^[^ ^1^ ^]^ To date, terrific efforts have been made in this field with the generation of 3D constructs capable of broadening knowledge on organ and tissue development and being used for translational applications.^[^ ^2^, ^3^ ^]^ While the precision and control over the generation of 3D in vitro constructs are being increasingly optimized thanks to progressively advanced technologies, there is a lack of information and investigation methods to predict the response of human cells to an engineered construct and subsequent interaction between the implant and host tissues. This is valid for any tissue type, including skeletal muscle (SkM).

In the last decade, several studies have been carried out for the in vivo replacement of large portions of SkM.^[^ ^4^, ^5^, ^6^, ^7^, ^8^ ^]^ Current reconstructive options for extensive damage to SkM tissue or congenital SkM malformations include autologous muscle grafts and intramuscular injection of progenitor cells.^[^ ^9^ ^]^ However, these therapeutic approaches are limited by many factors, such as the restricted availability of donor tissues (especially in pediatric patients), graft failure, donor site morbidity, and difficulties in regulating cell activity, which must be adequate and guided toward regeneration.^[^ ^9^ ^]^ Moreover, SkM defects and damages are often accompanied by injury to the neuronal network directly connected to the muscle tissue, leading to atrophy and permanent functional disability of the denervated muscle, also after treatment.^[^ ^10^ ^]^

Several strategies have been developed in recent years to promote myogenesis and neuromuscular junction (NMJ) regeneration even after large volumes of muscle loss. Some tissue engineering approaches have included the use of customized scaffolds with controlled release of neurotrophic molecules,^[^ ^11^, ^12^ ^]^ also in combination with exercise,^[^ ^4^, ^11^ ^]^ to promote host‐cell migration and/or invasion. It is known that scaffolds obtained through the decellularization of SkM (dSkM) possess intrinsic myogenic^[^ ^5^, ^6^, ^13^ ^]^ and neurotrophic properties,^[^ ^14^ ^]^ and when implanted in vivo as devices (i.e., without pre‐seeding of muscular cells) can integrate with the host tissue in animal models and stimulate nerve sprouting, while simultaneously attracting myogenic progenitors inside the scaffold itself^[^ ^13^ ^]^ with consequent SkM and NMJ regeneration.^[^ ^6^ ^]^ Importantly, apart from evidence in animal models, it has been demonstrated that the implantation of decellularized scaffolds as devices (i.e., no pre‐seeded with myogenic cells) could promote a certain degree of muscle recovery in patients affected by volumetric muscle loss.^[^ ^15^ ^]^ However, this and other studies^[^ ^16^, ^17^ ^]^ suggest that the use of a decellularized scaffold as a medical device is not sufficient to guarantee full tissue recovery. A major challenge that hinders the complete functional integration of implanted scaffolds is the achievement of adequate and timely innervation by the host peripheral nervous system.^[^ ^18^ ^]^ The use of dSkM pre‐seeded with muscular cells (i.e., recellularized dSkMs, here defined as rSkMs) has been shown to be the most efficient solution to repair diaphragmatic hernia defects in animal models, as it can already provide a well‐organized and functioning cytostructure^[^ ^19^ ^]^ that can support the regeneration of the innervated SkM. However, the use of rSkM in patients affected by congenital or traumatic volumetric muscle loss conditions has never been reported, and no methods are currently available to predict the human cell response toward a recellularized construct in terms of myogenic regeneration, neuronal integration, and functional NMJ formation. Indeed, at present, these humanized tissue‐engineered constructs must be necessarily implanted in vivo in a xenogeneic environment to evaluate their integration properties, increasing the limiting aspects related to correctly predicting human responses.^[^ ^20^, ^21^ ^]^

Organoids derived from both embryonic stem cells or human induced pluripotent stem cells (hiPSCs) have been recently derived to enable the production of in vitro models containing isogenic muscular and neuronal compartments arranged into spheroids ‐ to which we refer here as neuromuscular organoids (NMOs).^[^ ^22^, ^23^, ^24^, ^25^ ^]^ The neuronal and muscular components in NMOs develop in parallel, self‐compartmentalize, and interact to form functional NM networks with the formation of NMJs and consequent myofiber contraction.^[^ ^22^, ^24^, ^26^, ^27^, ^28^ ^]^ Such NMOs have been therefore used to model and study in vitro the human NM system in health and disease.^[^ ^22^, ^23^, ^24^, ^25^ ^]^ We also recently derived tissue‐engineered NMOs by the direct seeding of hiPSCs onto dSkMs, which offered a 3D extracellular environment able to promote the maturity of the NMOs and to mimic in vitro Duchenne Muscular Dystrophy myogenic phenotypes.^[^ ^29^ ^]^ Moreover, Shin and colleagues have recently shown that SkM organoids derived from hiPSCs could be used to mimic and study in vitro myogenesis and regeneration of the human SkM upon acute damage with cardiotoxin.^[^ ^24^ ^]^ Notably, the described SkM organoids also possessed a neuronal compartment.^[^ ^24^ ^]^ Due to recent reports, the NMO technology is now considered a valuable tool to study and predict responses of the human NM system to damages and/or treatments.

Here we aim to study in vitro the human NM system response toward an engineered SkM construct that could be applicable in regenerative medicine. To do so, we combined NMO technology with a SkM tissue engineering approach, to develop a graft‐host SkM assembloid model where the interaction of engineered constructs and the human NM system and their response to acute damage could be studied. Specifically, assembloids are in vitro models that combine two or more organoids, spheroids, or cultured cell types to recapitulate the structural and functional properties of an organ or a system.^[^ ^30^ ^]^ We used dSkM‐based products as SkM tissue‐engineered donor grafts and NMOs as recipient compartments to derive the engineered assembloids. We found that pre‐seeding human muscle progenitors (hMPCs) within the scaffold (recellularized SkM, rSkM) promoted NMO host‐cell invasion of the engineered constructs, when compared to scaffolds provided as device (dSkM is considered without pre‐seeded hMPCs), with functional integration and NMJ formation. Furthermore, we subjected NMO‐rSkM assembloids to acute damage via cardiotoxin treatment and we found that the assembloid was able to regenerate, rescuing the SkM function and partially the NMJs.

Implications of our research are significant and may contribute to advancements in tissue engineering and regenerative medicine of the human NM system, opening new perspectives for the translation of engineered SkM grafts to patients. Moreover, the possibility of faithfully analyzing the interaction between the human NM system and engineered SkM and vice versa represents an intriguing biological aspect that can increase our knowledge of the human NM system regeneration.

## 2 Results

### 2.1 Assembloids can be Derived by Combining dSkM Scaffolds or rSkM Tissue‐Engineered Constructs and NMOs

**Figure 1:**
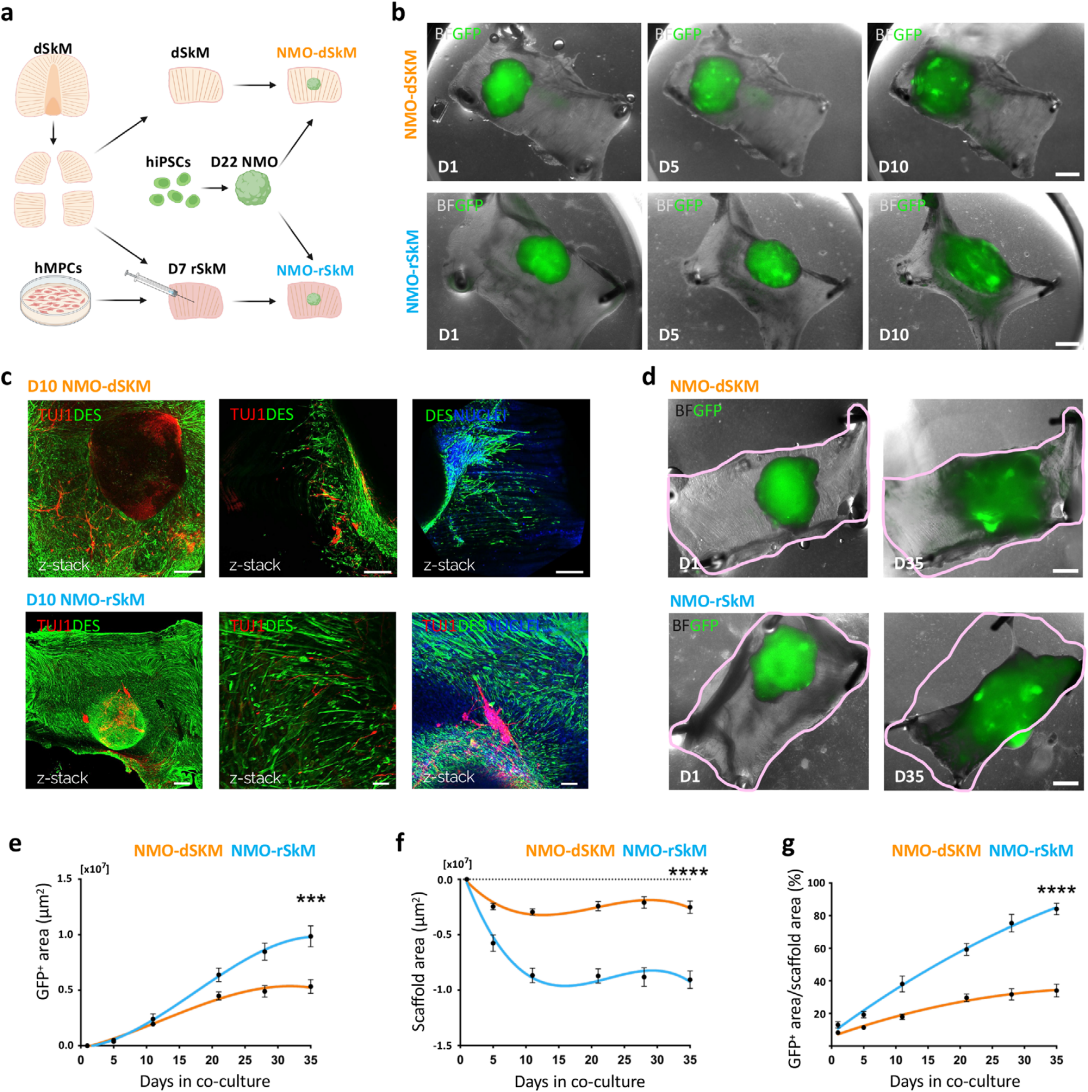
NMOs integrate within dSkMs and rSkMs to form graft‐host SkM assembloids. a) Schematic illustration showing the strategy used for co‐culturing NMOs and dSkMs (NMO‐dSkM, orange) or rSkMs (NMO‐rSkM, light blue). NMOs were derived from hiPSCs upon 22 days (D22 NMO) of NM differentiation. rSkMs were generated upon hMPCs injection within dSkM 7 days (D7 rSkM) before NMOs seeding. Acronyms list: dSkM: decellularized skeletal muscle; hMPCs: human muscle progenitor cells; hiPSCs: human induced pluripotent stem cells; D22 NMO: day 22 neuromuscular organoids; D7 rSkM: day 7 recellularized skeletal muscle construct; NMO‐dSkM: construct obtained from NMO combined with dSkM; NMO‐rSKM: construct obtained from NMO combined with rSkM. b) Representative brightfield (BF) and fluorescence stereomicroscope images showing the progression of GFP‐NMOs at day 1 (D1), day 5 (D5) and day 10 (D10) after seeding onto dSkM (NMO‐dSkM, upper panels) or onto rSkM (NMO‐rSkM, lower panels). Scale bars, 1 mm. c) Upper left and middle panels, representative Z‐stack confocal immunofluorescence images of whole mount NMO‐dSkM after 10 days of co‐culture (D10 NMO‐dSkM) stained for DESMIN (green) and TUJ1 (red). Upper right panel, representative Z‐stack confocal immunofluorescence image of whole mount NMO‐dSkM after 10 days of co‐culture (D10 NMO‐dSkM) stained for DESMIN‐positive cells (green) that invade the dSkM. Nuclei are counterstained with Hoechst (blue). Scale bars, 200 µm. Lower panel, representative Z‐stack confocal immunofluorescence images of whole mount NMO‐rSkM after 10 days of co‐culture (D10 NMO‐rSkM) stained for DESMIN (green) and TUJ1 (red). Right panel, nuclei are counterstained with Hoechst (blue). Scale bars, 500 µm (left), 50 µm (center), 100 µm (right). d) Representative brightfield (BF) and fluorescence stereomicroscope images showing the expansion of GFP‐NMOs after 1 day (D1) and 35 days (D35) from seeding onto dSkM (NMO‐dSKM) or rSkM (NMO‐rSKM). The pink line is used to track the morphological changes of the overall construct shape at the two co‐culture time points. Scale bar, 1 mm. e) NMO area variation (µm^2^) over culture time (days), expressed as difference between GFP^+^ area at day n and GFP^+^ area at day 1 of co‐culture. Data are shown as mean ± SEM of *n* > 9 independent replicates. Statistical significance determined using Mann‐Whitney U test; ***p = 0.0008 on day 35 (0.99 × 10^7^ ± 0.09 × 10^7^ µm^2^ versus 0.53 × 10^7^ ± 0.06 × 10^7^ µm^2^). f) Scaffold area variation (µm^2^) over culture time (days), expressed as difference between scaffold area at day n and scaffold area at day 1 of co‐culture. Data are shown as mean ± SEM of n>9 independent replicates. Statistical significance determined using Mann‐Whitney U test; ****p < 0.0001 on day 35 (−0.91 × 10^7^ ± 0.07 × 10^7^ µm^2^ vs −0.25 × 10^7^ ± 0.06 × 10^7^ µm^2^). g) Ratio between NMO area and scaffold area, expressed in percentage. Data are shown as mean ± SEM of *n* > 9 independent replicates. Statistical significance determined using Mann‐Whitney U test; ****p < 0.0001 on day 35 (84.02 ± 3.52% vs 34.04 ± 3.92%).

With the aim to investigate the integration between the NM system of human origin and engineered muscle constructs, we initially cultured NMOs derived from human induced pluripotent stem cells (hiPSCs)^[^ ^29^ ^]^ with dSkM scaffolding biomaterials (named NMO‐dSkM assembloids) or with 7‐day rSkM constructs^[^ ^31^ ^]^ (named NMO‐rSkM assembloids; **Figure** **1**a). Throughout the entire study, we alternatively used wild‐type hiPSCs and hiPSCs constitutively expressing green fluorescent protein (GFP) under the control of 3‐phosphoglycerate kinase promoter to derive NMOs (Figure S1, Supporting Information). We used GFP‐NMOs to easily follow the localization of cells derived from NMOs within our engineered SkM constructs, while wild‐type NMOs were used for dedicated imagining analysis where the GFP signal could interfere – including Fluo‐4 live imaging and immunofluorescence analysis. Upon selection and sorting of GFP‐hiPSCs when necessary (Figure S1a,b, Supporting Information), cells were characterized for pluripotency‐related markers (Figure S1c–e, Supporting Information) and used to produce self‐assembled spheroidal NMOs (Figure S2, Supporting Information). NMOs were derived using a small molecule‐based differentiation protocol in the presence of Matrigel droplets until day 22 (D22) from the beginning of the differentiation period^[^ ^29^ ^]^ (Figure S2a, Supporting Information). At first, we confirmed that GFP expression was still present at this stage of NMO differentiation (Figure S2b, Supporting Information). We found that D22 NMOs showed muscular and neuronal compartmentalization, with a neuronal network that reached elongated desmin (DES)‐expressing cells (Figure S2c,d, Supporting Information). As shown by immunofluorescence analysis, D22 NMOs also possess both paired box protein 7 (PAX7)‐expressing stem cells and myogenic committed myogenin (MYOG)‐positive progenitor cells, associated to myofibers that express embryonic myosin heavy chain (eMHC) in contact with laminin (LAM) deposited in the extracellular matrix (Figure S2e–h, Supporting Information).

To produce tissue‐engineered human SkM constructs, we used an already well‐characterized decellularization protocol to derive dSkM from the murine diaphragm,^[^ ^32^, ^33^ ^]^ which was used per se (dSkM) or recellularized (rSkM) with hMPCs isolated from healthy muscle biopsies.^[^ ^34^ ^]^ We already demonstrated that dSKM sustains hMPC engraftment, proliferation, and differentiation, generating 3D rSkM tissue‐engineered constructs suitable for in vivo implantation.^[^ ^19^ ^]^ Nevertheless, before being used to generate rSkM, we expanded and characterized hMPCs in culture (Figure S3a, Supporting Information) confirming that they possess high cell rates of CD56 (94.18% ± 2.22%) and MYOD (99.22% ± 0.17%) expressing cells and low rates of MYOG (3.86% ± 0.61%) positive cells (Figure S3b–d, Supporting Information). In agreement with previous studies,^[^ ^31^, ^34^ ^]^ a small proportion of TE7^+^ (0.93% ± 0.15%) fibroblasts were also identified in the primary hMPC culture (Figure S3e, Supporting Information). Finally, to confirm the myogenic ability of hMPCs, we evaluated and quantified the proliferation rate and the ability to form myotubes in vitro (Figure S3f,g, Supporting Information).

To understand the feasibility of the NMO/SkM‐engineered assembloids, we first performed short‐term experiments by seeding D22 GFP‐NMOs onto dSkM or rSkM, and by culturing the assembloids for 10 days (Figure 1b). In both cases (NMO‐dSkM and NMO‐rSkM assembloids), cells derived from NMOs integrated within the tissue‐engineered products, as shown by the sprouting of both neural and muscular components observed in whole mount samples immunostained for TUJ1^+^ neuronal projection and DES^+^ myogenic cells, respectively (Figure 1c). During the time of culture and at longer time points (35 days), we observed invasion of GFP‐expressing cells within the scaffolds and a differential gross appearance of NMO‐dSkM and NMO‐rSkM assembloids (Figure 1b,d; Figure S4a,b, Supporting Information). In particular, we found that both tissue‐engineered SkM and GFP‐NMO shapes were evidently remodeled in NMO‐rSkM after 35 days of co‐culture when compared to NMO‐dSkM assembloids at equivalent time points (Figure 1d). To quantify such observation, we monitored NMO‐rSkM and NMO‐dSkM cultures, and we measured over time (from day 0 to day 35) the area occupied by GFP^+^ cells (Figure 1e), the scaffold area variation (Figure 1f) and their ratio (Figure 1g). We confirmed that NMOs showed an improved ability to spread, remodel, and invade the scaffold when in contact with hMPC pre‐seeded constructs –, i.e., NMO‐rSkM assembloids (Figure 1d–g). Interestingly, we also found that hMPCs had per se the ability to remodel the dSkM during the time of culture –, i.e., rSkM samples (Figure S4c,d, Supporting Information). Altogether these data demonstrate that both dSkM and rSkM can be invaded by human NM system derived from the NMO in vitro, generating graft‐host SkM assembloids.

### 2.2 Pre‐Seeding of hMPCs into dSkMs Promotes Myogenesis and Muscle Functionality in NMO‐rSkM Assembloids

**Figure 2:**
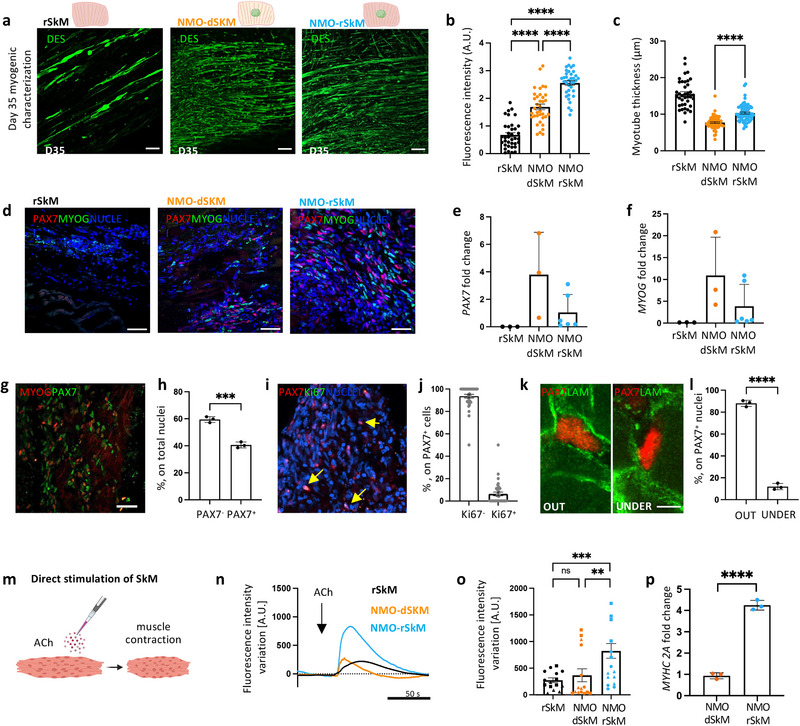
Myogenesis and muscle functionality are improved in NMO‐rSkM assembloids. a) Representative Z‐stack confocal immunofluorescence images of whole mount rSkM (left panel), NMO‐dSkM (center panel) or NMO‐rSkM (right panel) after 35 days (D35) of co‐culture, stained for DESMIN (green). Scale bars, 100 µm. b) Quantification of corrected total fluorescence intensity normalized for background signal performed on DESMIN immunofluorescence staining. Data are shown as mean ± SEM of 36 ROIs per condition (three images per condition, for each image 12 ROIs were selected and quantified). Statistical significance was determined using Mann‐Whitney U test; ****p < 0.0001. Statistical results are reported in Table S1 (Supporting Information). c) Quantification of DESMIN+ myotube cross‐section (thickness) performed on whole mount immunofluorescence images of rSkM, NMO‐dSkM and NMO‐rSkM after 35 days (D35) of culture. Data are shown as mean ± SEM of >100 myotube cross sections, measured on 3 independent samples per condition; statistical significance determined using Mann‐Whitney U test; ****p < 0.0001. Statistical results are reported in Table S2 (Supporting Information). d) Representative confocal immunofluorescence image of rSkM, NMO‐dSkM and NMO‐rSkM cross‐sections, stained for PAX7 (red) and MYOG (green). Nuclei are counterstained with Hoechst (blue). Scale bar, 50 µm. e) *PAX7* gene expression in rSkMs, NMO‐dSkMs and NMO‐rSkMs. Data are normalized to housekeeping *B2‐microglobulin* gene expression and shown as fold change over NMO. Data are shown as mean ± s.d. of three independent biological replicates for rSkM and NMO‐dSkM and of six independent biological replicates for NMO‐rSkM. Statistical results are reported in Table S3 (Supporting Information). f) *MYOG* gene expression in rSkMs, NMO‐dSkMs and NMO‐rSkMs. Data are normalized to housekeeping B2‐microglobulin gene expression and shown as fold change over NMO. Data are shown as mean ± s.d. of three independent biological replicates for rSkM and NMO‐dSkM and of 6 independent biological replicates for NMO‐rSkM. Statistical results are reported in Table S4 (Supporting Information). g) Representative Z‐stack confocal immunofluorescence image NMO‐rSkM cross‐sections at day 35 of co‐culture stained for MYOG (red) and PAX7 (green). Nuclei are counterstained with Hoechst (grey). Scale bars, 50 µm (a). h) Quantification of PAX7^+^ and PAX7^−^ cells on total cells in untreated samples and expressed in percentage. Data are shown as mean ± s.d. of n>10 images, taken on three independent biological replicates. Statistical significance was determined using Mann‐Whitney U test; ***p = 0.0005. i) Representative Z‐stack confocal immunofluorescence image of NMO‐rSkM cross‐sections at day 35 of co‐culture stained for PAX7 (red) and Ki67 (green). Nuclei are counterstained with Hoechst (blue). Scale bar, 50 µm. j) Quantification of PAX7^+^Ki67^+^ and PAX7^+^Ki67^−^ cells on total PAX7^+^ cells in untreated samples and expressed in percentage. Data are shown as mean ± SEM of *n *> 10 images, taken on 2 independent biological replicates. k) Representative confocal immunofluorescence image NMO‐rSkM cross‐sections at day 35 of co‐culture showing different localization of PAX7 (red) out (left panel) or under (right panel) basal lamina (LAM, green). Scale bars, 10 µm. Single channels and nuclei staining are reported in Figure S5i (Supporting Information). l) Quantification of PAX7+ localized out or under the basal lamina on total PAX7^+^ cells in untreated samples and expressed in percentage. Data are shown as mean ± s.d. of *n* > 10 images, taken on three independent biological replicates. Statistical significance was determined using Mann‐Whitney U test; ****p < 0.0001. m) Schematic illustration showing the strategy used to assess muscle compartment functionality in response to exogenous ACh administration. n) Representative quantification of mean normalized fluorescence intensity variation registered during live imaging analysis of D35 rSkM, NMO‐dSkMs and NMO‐rSkMs, stimulated with ACh. Data are shown as mean of 9 ROIs, 3 ROIs were selected from each of 3 independent replicates; measurements with error bars are reported in Figure S6a–c (Supporting Information). Dotted line corresponds to the baseline equal to 0. o) Contraction quantification of rSkM, NMO‐dSkM or NMO‐rSkM at D35 upon exogenous ACh administration expressed as maximum peak of fluorescence revealed via live imaging analysis. Quantifications were performed on 3 independent biological replicates, identified by different symbol shapes in the graph. Each independent sample was divided into 5 ROIs and each point represents a single ROI. Statistical significance was determined using Mann‐Whitney U test; ns, not statistically significant, **p = 0.0066, ***p = 0.0001. Statistical results are reported in Table S5 (Supporting Information). p) *MYHC 2A* gene expression in NMO‐dSkMs and NMO‐rSkMs. Data are normalized to MYHC gene expression and shown as fold change over NMO‐dSkMs. Data are shown as mean ± s.d. of three independent biological replicates. Statistical significance was determined using unpaired t‐test; ****p < 0.0001.

It remains a matter of discussion in the field whether the pre‐seeding of tissue‐engineered constructs with cells could promote the integration of the graft with recipient cells. Toward this aim, we compared NMO‐dSkM and NMO‐rSkM assembloids after 35 days of culture to understand whether the pre‐seeding of hMPCs into dSkM could promote the integration of NMOs and overall myogenesis and muscle functionality of our in vitro assembloid model. As controls, rSkMs were included in the analysis to monitor sample behavior in the absence of NMO contribution. First, we performed morphometric analysis to evaluate the presence, localization, and organization of differentiated myogenic cells within the samples. We confirmed the invasion of the scaffolds by seeded cells in all samples, as shown by nuclei and laminin immunofluorescence analysis (Figure S4e, Supporting Information). Despite all samples showed elongated DES^+^ myogenic cells, we found that cells were extensively localized within the NMO‐rSkM assembloids, identified as discrete bundles into NMO‐dSkM assembloids, and as fewer cells within the rSkM samples (**Figure** **2**a,b; Figure S4f, Supporting Information). Accordingly, with the expected developmental stages of the myotubes (i.e., from adult hMPCs or fetal/neonatal NMOs), we also found that rSkMs displayed statistically significant thicker myotubes (15.53 ± 0.63 µm) when compared to NMO‐dSkM and NMO‐rSkM assembloids, with the presence of many but thin myotubes in the former (7.71 ± 0.21 µm), and many and significantly thicker myotubes in the latter (10.32 ± 0.26 µm; Figure 2a,c).

Apart from terminally differentiated myogenic cells, we also evaluated whether rSKMs, NMO‐dSkMs, and NMO‐rSkMs could possess muscle stem cells (PAX7‐expressing cells) and myogenic committed cells (expressing MYOG) after 35 days of culture. Immunofluorescence analysis showed that both NMO‐dSkM and NMO‐rSkM assembloids possess both stem and committed myogenic cells (Figure 2d; Figure S5, Supporting Information). The absence of PAX7‐expressing cells and the small number of MYOG‐expressing cells in rSkMs, strongly suggested that NMOs are the major contributor to stem and committed myogenic cell populations revealed within NMO‐dSkM and NMO‐rSkM (Figure 2d–f; Figure S5a–e, Supporting Information). Accordingly, with the stage of myogenic differentiation observed, we also identified MYOD‐expressing cells using immunofluorescence analysis (Figure S5f, Supporting Information). Since after 35 days of culture the NMO‐rSkM assembloids presented a relevant percentage of PAX7^+^ cells (40% ± 2.2%, Figure 2g,h), we further characterized them. More than 90% of PAX7^+^ cells were not actively proliferating, and only 6.28% ± 1.66% co‐expressed the proliferation marker Ki67 (Figure 2i,j). Interestingly, PAX7^+^ cells were found in well‐defined anatomical positions, with ≈12% of PAX7^+^ cells located beneath the basal lamina of the muscle fibers (Figure 2k,l; Figure S5g–i, Supporting Information), which defines the specific anatomical position of satellite cells.^[^ ^35^, ^36^ ^]^ Once confirmed the presence of SkM cells in all the samples, we then evaluated the ability of these models to contract when stimulated with acetylcholine (ACh) by quantifying fluorescence intensity variation associated with muscle contraction during live imaging analysis (Figure 2m). Recellularized SkM samples, mainly composed of sparse mature myotubes within the  scaffolding extracellular matrix (Figure S4f, Supporting Information), were able to respond to the exogenous ACh administration with a contraction intensity variation comparable to that of NMO‐dSkM assembloids (273.65 ± 43.79 A.U. versus 366.56 ± 121.45 A.U.), which oppositely possess more but smaller myotubes (Figure 2n,o; Figure S6a,b (Supporting Information) Video S1, Supplementary Video 1). On the contrary, NMO‐rSkMs responded with significantly higher contraction to ACh administration when compared to the other samples (823.65 ± 138.28 A.U.; Figure 2n,o; Figure S6a–c (Supporting Information) and Supplementary Video S1, Supplementary Video 1). This is in line with the presence of myotube contributions from both adult MPCs and NMOs. Accordingly, the expression of the myosin heavy chain 2A gene (*MYHC 2A*)^[^ ^37^ ^]^ was significantly upregulated in NMO‐rSkMs, when compared to NMO‐dSkMs (Figure 2p).

These results confirm the muscular integration of NMOs within tissue‐engineered constructs to form graft‐host SkM assembloids and demonstrate that the pre‐seeding of hMPCs within dSkM promotes myogenesis and muscle functionality in NMO‐rSkM.

### 2.3 NMO‐rSkM Assembloids Possess Functional NMJs after 35 Days of Co‐Culture

**Figure 3:**
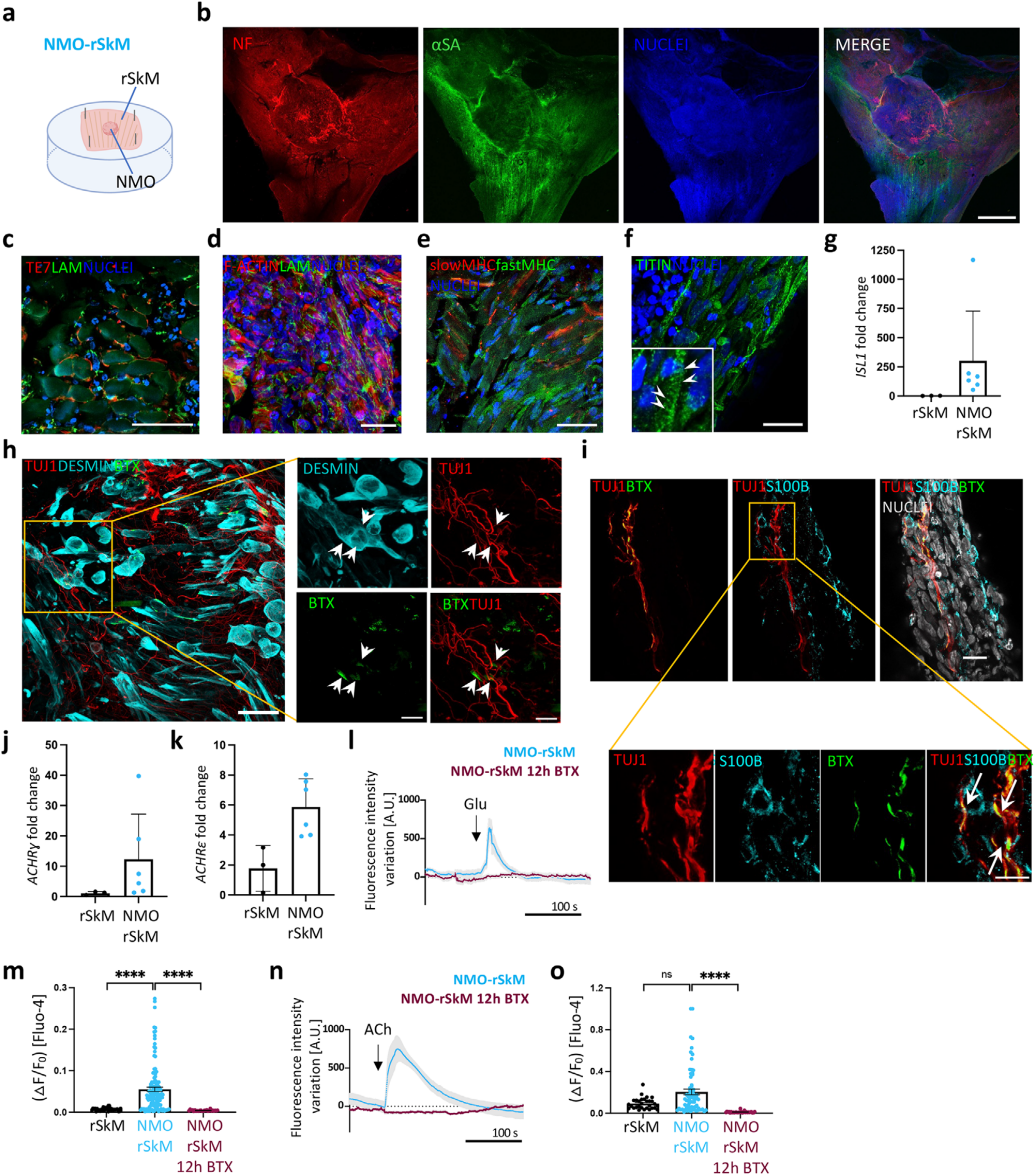
NMO‐rSkM assembloids possess functional NMJs after 35 days of co‐culture. a) Schematic illustration showing a representation of NMO‐rSkM co‐culture setup. b) Representative Z‐stack confocal immunofluorescence images of whole mount NMO‐rSkMs stained for NEUROFILAMENT (NF, red) and alpha‐SARCOMERIC ACTININ (αSA, green). Nuclei are counterstained with Hoechst (blue). Scale bar, 1 mm. c) Representative Z‐stack confocal immunofluorescence image of NMO‐rSkM cross‐sections stained for TE7 (red) and LAMININ (green). Nuclei are counterstained with Hoechst (blue). Scale bar, 50 µm. d) Representative Z‐stack confocal immunofluorescence image of NMO‐rSkM cross‐sections stained for F‐actin (red) and LAMININ (green). Nuclei are counterstained with Hoechst (blue). Scale bar, 20 µm. e) Representative Z‐stack confocal immunofluorescence image of NMO‐rSkM cross‐sections stained for slow MYOSIN HEAVY CHAIN (MHC, red) and fast MHC (green). Nuclei are counterstained with Hoechst (blue). Scale bar, 50 µm. f) Representative Z‐stack confocal immunofluorescence image of NMO‐rSkM cross‐sections stained for TITIN (green). Nuclei are counterstained with Hoechst (blue). Scale bar, 20 µm. Inset shows a higher magnification, evidencing sarcomeric organization of myofiber cytoskeleton highlighted by arrowheads. g) *ISL1* gene expression in rSkMs and in NMO‐rSkMs. Data are normalized to housekeeping *B2‐microglobulin* gene expression and shown as fold change over rSkM. Data shown as mean ± s.d. of three independent biological replicates for rSkM and of six independent biological replicates for NMO‐rSkM. Mann‐Whitney U test; ns, not statistically significant; *p = 0.0238. h. Representative Z‐stack confocal immunofluorescence image of NMO‐rSkM cross‐sections stained for TUJ1 (red), DESMIN (cyan) and α‐bungarotoxin^+^ (BTX) regions (green). Nuclei are counterstained with Hoechst (blue). Higher magnification of Figure 3g. is shown in h) White arrows indicate putative NMJ where signals colocalized. Scale bars, 50 µm (g) and 10 µm (h). i) Representative Z‐stack confocal immunofluorescence image of NMO‐rSkM cross‐sections stained for TUJ1 (red), BTX (green) and S100β (cyan). Nuclei are counterstained with Hoechst (grey). Scale bar, 20 µm (upper panel) and 10 µm (lower panel). White arrows indicate putative NMJ where signals colocalized. j. *ACHRɣ* gene expression in rSkMs and in NMO‐rSkMs. Data are normalized to housekeeping *B2‐microglobulin* gene expression and shown as fold change over rSkM. Data shown as mean ± s.d. of three independent biological replicates for rSkM and of six independent biological replicates for NMO‐rSkM. Mann‐Whitney U test; *p = 0.0476. k) *ACHRε* gene expression in rSkMs and in NMO‐rSkMs. Data are normalized to housekeeping *B2‐microglobulin* gene expression and shown as fold change over rSkM. Data shown as mean ± s.d. of three independent biological replicates for rSkM and of six independent biological replicates for NMO‐rSkM. Mann‐Whitney U test; *p = 0.0238. l, m) Representative quantification of mean normalized fluorescence intensity variation registered during live imaging analysis of NMO‐rSkMs or NMO‐rSkMs treated for 12 h with BTX and stimulated with Glu (l) or ACh (n). Data are shown as mean ± SEM of 15 ROIs (5 ROIs per sample, from three independent biological replicates) for untreated NMO‐rSkMs (light blue line). Data are shown as mean ± SEM of 10 ROIs (5 ROIs per sample, from two independent biological replicates) for NMO‐rSkMs treated overnight with BTX (crimson line). Dotted lines correspond to the baseline equal to 0. Statistical results are reported in Table S6 (Supporting Information). n, o) Quantification of calcium peak amplitude (ΔF/F0) detected with Fluo‐4 live imaging analysis of rSkM (*n* = 2), NMO‐rSkM (*n* = 4) or NMO‐rSkM after BTX treatment (*n* = 2) after Glu (m) or ACh (o) administration. The fluorescence intensity peak (F) during stimulation was measured and normalized to the baseline fluorescence intensity registered before neurotransmitter stimulation (F0). Mann‐Whitney U test; ns, not statistically significant; ****p < 0.0001. Statistical results are reported in Table S7 (Supporting Information).

Based on the above results, we focused our next studies on NMO‐rSkM (**Figure** **3**a). One of the most limiting aspects in the in vivo application of muscle implants is the achievement of adequate and timely innervation by the host peripheral nervous system.^[^ ^18^ ^]^ For this reason, we analyzed the presence of functional NMJs in NMO‐rSkM assembloids. From a general point of view, day 35 NMO‐rSkM assembloids displayed strong and wide neurofilament (NF) positive axons which spread from the central body of the NMO to also distal parts of alpha‐sarcomeric actin (αSA)‐expressing cells (Figure 3b). The NMO‐rSkM assembloids appeared well integrated as shown by scanning electron microscopy and histological staining (Figure S6d–f, Supporting Information). Moreover, together with the expression of myogenic key transcriptional factors (Figures S5 and S7a, Supporting Information), we found the presence of supporting muscle cells such as TE7^+^ fibroblasts (Figure 3c), deposition of basal lamina (Figure 3d) and the presence of muscular cells expressing proteins essential for their contraction, such as slow and fast MHC and sarcomeric TITIN (Figure 3e,f). Based on the presence of a neuronal compartment that extends toward the muscle (Figure S7b, Supporting Information), we further characterized it by combining immunofluorescence and gene expression analysis. In particular, we found the presence of neural progenitors co‐expressing paired Box 6 nuclear factor (PAX6) and SRY‐box transcription factor 2 (SOX2),^[^ ^38^ ^]^ while the expression of the transcription factors LIM/homeodomain family of transcription factors ISLET1, motor neuron (MN)‐determinant homeobox gene MNX1 (i.e., HB9), and choline acetyltransferase (CHAT),^[^ ^39^ ^]^ strongly suggested the presence of MNs (Figure 3g; Figure S7c–e, Supporting Information).

Accordingly, we also identified juxtaposed pre‐ and post‐synaptic elements of the NMJs in anatomically expected positions (Figure 3h; Figure S7f, Supporting Information). Moreover, in close contact between TUJ1^+^ neuronal protrusions and alpha‐bungarotoxin (BTX^+^) signal, we identified the presence of S100B^+^ cells (Figure 3i; Figure S7g, Supporting Information), suggesting the presence of terminal Schwann cells.^[^ ^22^ ^]^ All these data are also supported by the expression of different isoforms of ACh receptors (*ACHRγ* and *ACHRε*), known to be associated with the maturation of the NMJs,^[^ ^40^ ^]^ that were statistically over‐expressed in NMO‐rSkMs when compared to rSkMs (Figure 3j,k).

To test whether the identified NMJs were also functional, we stimulated NMO‐rSkMs with glutamate (Glu) and evaluated the muscle contraction. In vivo, Glu stimulates the MN to release ACh at the NMJ, inducing muscle contraction.^[^ ^40^ ^]^ In agreement, we found that Glu‐mediated stimulation resulted in NMO‐rSkM assembloid contraction and in a significant increase of calcium spikes in the muscular compartment, thus indicating the presence of functional NMJs (Figure 3l,m; Figure S7h–j (Supporting Information) and Video S2, Supplementary Video 2). Importantly, calcium transients were not revealed in rSkM samples, which do not possess a neuronal compartment and thus do not have NMJs, indicating the specificity of action of the Glu at the muscular level via the neuronal stimulation (Figure 3m). In agreement, Glu‐mediated muscle contraction and calcium response were abolished upon treatment with BTX, known to block the NMJ at the postsynaptic level^[^ ^40^ ^]^ (Figure 3l,m; Figure S7i, Supporting Information). As control of the physiological activity of the muscle compartment in our samples, we also supplemented ACh to reveal signals derived upon direct simulation of AChRs situated on myofibers. As expected, the addition of exogenous ACh was able to induce a positive response in terms of contraction and calcium transient in all the analyzed samples, including rSkMs (Figure 3n,o; Figure S7j (Supporting Information) and Video S3, Supplementary Video 3). In agreement, AChR block with BTX led to the loss of NMO‐rSkM contraction and calcium transients after stimulation with ACh, highlighting the physiological behavior of the system (Figure 3n,o; Figure S7j (Supporting Information) and Videos S2 and S3, Supplementary Video 2 and 3).

Taken together, these data indicate a functional NM system integration between the NMOs and tissue‐engineered muscle constructs in NMO‐rSkM assembloids.

### 2.4 NMO‐rSkM Assembloids Possess Muscle Stem Cells and Model Regeneration Events Upon Acute Damage

To define the integration between the NMO and the rSkM (i.e., NMO‐rSkM) as definitely functional and potentially long‐lasting, it is necessary to verify that the assembloids could respond to acute injury with regeneration. A relevant body of studies demonstrated the key role of PAX7‐expressing cells for proper SkM regeneration.^[^ ^35^, ^36^ ^]^ We have previously shown that after 35 days of culture, NMO‐rSkM assembloids present a high proportion of satellite cells, most of which do not express the Ki67 marker (Figure 2d–l).

**Figure 4:**
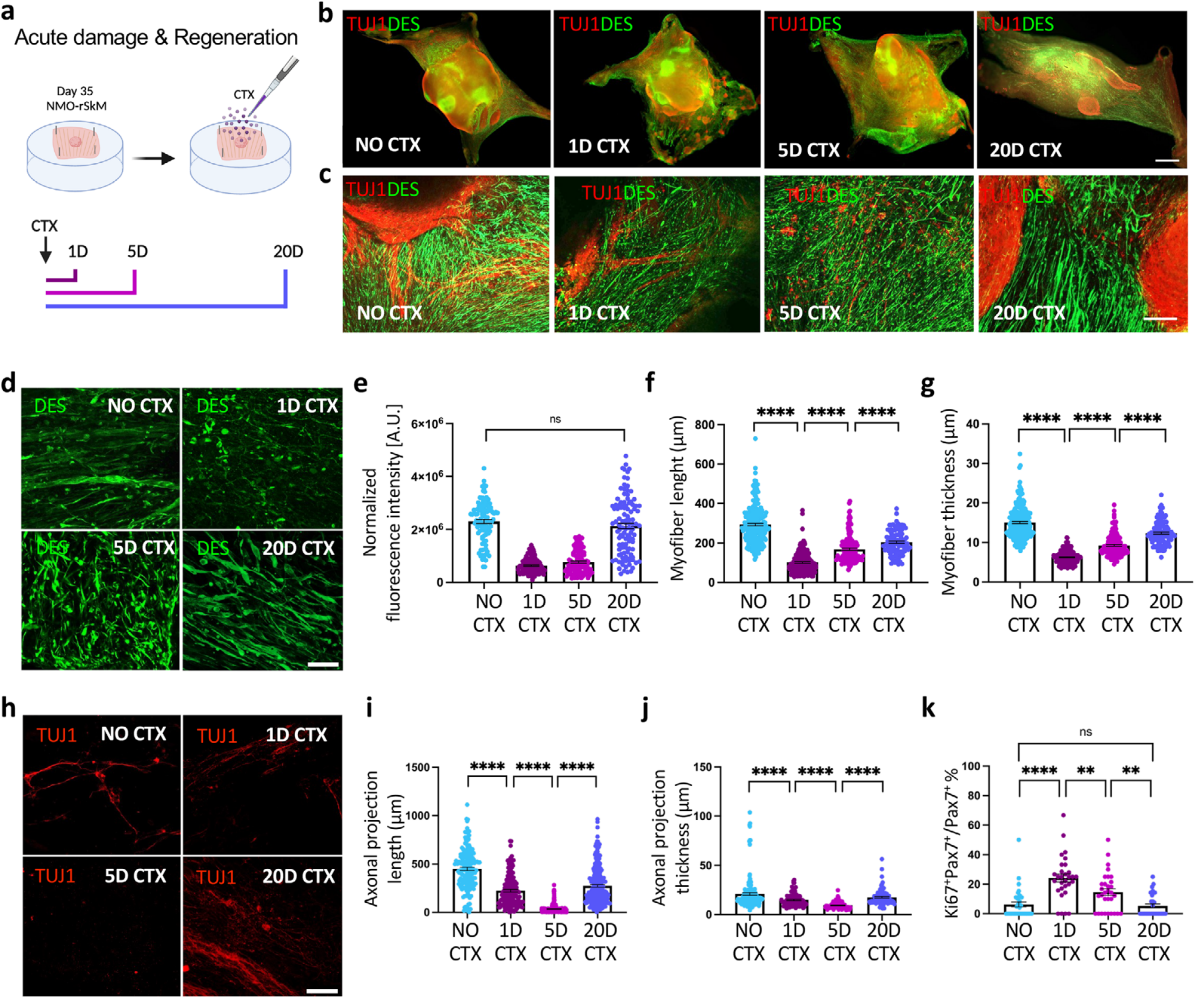
NMO‐rSkM assembloids possess muscle stem cells and model degeneration and regeneration events upon acute damage. a) Schematic illustration showing the experimental procedure and timeline used to investigate the regenerative ability of NMO‐rSkMs. b) Representative stereomicroscope images showing whole mount immunofluorescence staining for TUJ1 (red) and DESMIN (green) of NMO‐rSkMs at day 35 of co‐culture not treated with cardiotoxin (no CTX) or day 1, day 5 and day 20 after CTX treatment. Scale bar, 1 mm. c) Representative Z‐stack confocal immunofluorescence images for TUJ1 (red) and DESMIN (green), showing a higher magnification of the corresponding stereomicroscope images. Scale bars, 200 µm. d) Representative Z‐stack confocal immunofluorescence images of NMO‐rSkMs at D35 of co‐culture not treated with cardiotoxin (no CTX) or 1 day, 5 days and 20 days after CTX treatment and stained in whole mount for DESMIN (green). Scale bar, 50 µm. e) Quantification of total fluorescence intensity normalized for background signal performed on DESMIN immunofluorescence staining. Data are shown as mean ± SEM of 36 ROIs per biological sample per each condition (*n* = 3). Statistical significance was determined using Mann‐Whitney U test; ns, not statistically significant. Statistical results are reported in Table S8 (Supporting Information). f) Quantification of myofiber length performed on DESMIN immunofluorescence staining. Data are shown as mean ± SEM of >30 myofibers per sample per each condition (*n* = 3), measured on ≥ three images per condition. Statistical significance was determined using Mann‐Whitney U test; ****p < 0.0001. g) Quantification of myofiber thickness performed on DESMIN immunofluorescence staining. Data are shown as mean ± SEM of >30 myofiber cross‐sections per sample per each condition (*n* = 3), measured on ≥ three images per condition. Statistical significance was determined using Mann‐Whitney U test; ****p < 0.0001. Statistical results are reported in Table S10 (Supporting Information). h) Representative Z‐stack confocal immunofluorescence images of NMO‐rSkMs at day 35 of co‐culture not treated with cardiotoxin (no CTX) or 1 day, 5 days and 20 days after CTX treatment and stained in whole mount for TUJ1 (red). Scale bar, 50 µm. i) Quantification of axonal projection length performed on TUJ1 immunofluorescence staining. Data are shown as mean ± SEM of >50 axonal projections per sample per each condition (*n* = 3), measured on ≥ three images per condition. Statistical significance was determined using Mann‐Whitney U test; ****P<0.0001. Statistical results are reported in Table S11 (Supporting Information). j) Quantification of axonal projection thickness performed on TUJ1 immunofluorescence staining. Data are shown as mean ± SEM of >30 axonal cross‐sections per sample per each condition (*n* = 3), measured on ≥ three images per condition. Statistical significance was determined using Mann‐Whitney U test; ****p < 0.0001. Statistical results are reported in Table S12 (Supporting Information). k) Quantification of Ki67^+^ cells among PAX7^+^ cells in NMO‐rSkMs at day 35 of co‐culture not treated with cardiotoxin (no CTX) or 1 day, 5 days and 20 days after CTX treatment, expressed in percentage. Data are shown as mean ± SEM of ≥ 30 images, from two different biological samples, per each condition. Statistical results are reported in Table S13 (Supporting Information).

Based on these observations, and to verify the regenerative abilities of our assembloids, we injured 35‐day NMO‐rSkMs with cardiotoxin^[^ ^24^, ^41^ ^]^ (CTX) to damage muscle fibers and evaluate degeneration and self‐regeneration after 1, 5, and 20 days from injury (**Figure** **4**a). Thus, we initially used morphometric analysis of whole‐mount immunostained samples to qualitatively estimate the muscular and neuronal compartment organization before the damage, just after the injury, and at longer time points. Already after 1 day from CTX treatment, a decreased presence of elongated DES^+^ cells was evident, especially in correspondence with the area closer to the scaffold (Figure 4b,c). Upon 5 days from CTX injury, we observed a slight amelioration in the muscular compartment, which presented thin elongated DES^+^ cells, and a spotted and less extended TUJ1^+^ neuronal axon network (Figure 4b,c). Accordingly, the expression of eMHC confirmed the presence of newly formed myofibers after 5 days from the acute injury, indicating the beginning of muscle regeneration (Figure S8a, Supporting Information). Interestingly, a morphometric organization of the neuronal and muscular compartments closer to the one of an undamaged construct was reached 20 days after CTX administration (Figure 4b,c). Thus, we quantified the morphological changes observed upon injury and regeneration verifying that following CTX treatment in NMO‐rSkM assembloids the muscle compartment was clearly damaged after 1 and 5 days from the injury, but restored to pre‐CTX levels after 20 days, as shown by the quantification of the normalized intensity fluorescence of DES^+^ cells (Figure 4d,e). Accordingly, myotube principal parameters, such as length and thickness, followed the same dynamics, with a significant reduction the upon 1 day from damage and significant improvement after 5 and 20 days after injury (Figure 4f,g). Even though with delayed timing with respect to the muscular compartment, we found that also the neuronal network showed damage and regeneration upon CTX treatment, as shown by the quantification of the neuronal protrusion length and thickness of TUJ1^+^ cells 1, 5, and 20 days after CTX (Figure 4h–j). Finally, and accordingly with literature,^[^ ^36^, ^41^, ^42^ ^]^ we found that the structural regenerative phase of the assembloids was anticipated by a prominent change in the proportion of proliferating PAX7^+^ stem cells, which increased significantly in the first days after injury (24.20% ± 2.70% on day 1 and 14.72% ± 2.38% on day 5) returning to basal levels on day 20 (6.28% ± 1.66% in the untreated sample and 5.46% ± 1.40% on day 20; Figure 4k).

### 2.5 NMO‐rSkM Assembloids Display Functional Muscle Regeneration upon 20 Days from Acute Damage

**Figure 5:**
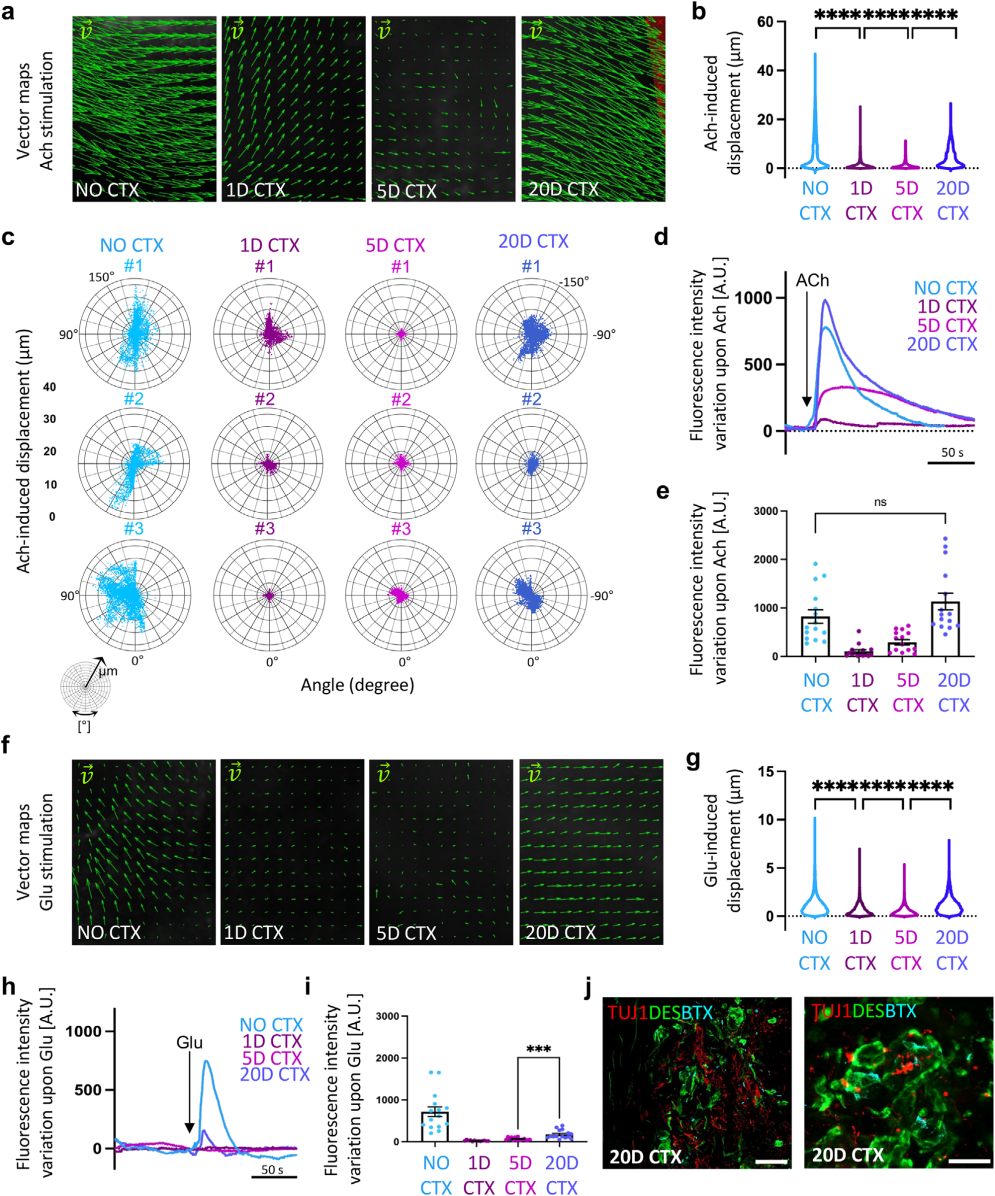
Functional muscle regeneration precedes neuronal and NMJ regeneration in NMO‐rSkM assembloids. a) Representative vector maps of displacements registered during contraction of NMO‐rSkMs at day 35 of co‐culture not treated with cardiotoxin (no CTX) or 1 day, 5 days and 20 days after CTX treatment when stimulated with ACh. b) Violin plot showing the maximum displacement of NMO‐rSkMs at day 35 of co‐culture not treated with cardiotoxin (no CTX) or 1 day, 5 days and 20 days after CTX treatment when stimulated with ACh. Each violin represents the mean of the displacement vectors obtained from 3 biological replicates per experimental condition (vector number analyzed ≥ 14951). Statistical significance was determined using one‐way ANOVA with Tukey's multiple comparisons test, ****P<0.0001. Statistical results are reported in Table S14 (Supporting Information). c) Representative polar charts showing quantification of the maximum displacement (µm) and of the displacement directionality (angle, degree) obtained with PIVlab analysis of NMO‐rSkMs at day 35 of co‐culture not treated with cardiotoxin (no CTX) or 1 day, 5 days and 20 days after CTX treatment when stimulated with ACh of three different assemblois (#) per each experimental condition. d) Representative quantification of mean normalized fluorescence intensity variation registered during contraction of NMO‐rSkMs at day 35 of co‐culture not treated with cardiotoxin (no CTX) or 1 day, 5 days and 20 days after CTX treatment when stimulated with ACh. For each curve, data are shown as the mean of 3 independent replicates. e) Contraction quantification of NMO‐rSkM at D35 not treated with cardiotoxin (no CTX) or 1 day, 5 days and 20 days after CTX treatment. Contraction was recorded upon exogenous ACh administration, and is expressed as the maximum peak of fluorescence revealed via live imaging analysis. Quantifications were performed on three independent biological replicates per condition. Each of the three samples was divided into 5 ROIs and every dot represents a single ROI. Statistical significance was determined using Mann‐Whitney U test; ns, not statistically significant. Statistical results are reported in Table S15 (Supporting Information). f) Representative vector maps of displacements registered during contraction of NMO‐rSkMs at day 35 of co‐culture not treated with cardiotoxin (no CTX) or 1 day, 5 days and 20 days after CTX treatment when stimulated with Glu. g) Violin plot showing the maximum displacement of NMO‐rSkMs at day 35 of co‐culture not treated with cardiotoxin (no CTX) or 1 day, 5 days and 20 days after CTX treatment when stimulated with Glu. Each violin represents the mean of the displacement vectors obtained from three biological replicates per experimental condition (vector number analyzed ≥ 15227). Statistical significance was determined using one‐way ANOVA with Tukey's multiple comparisons test, ****p < 0.0001. Statistical results are reported in Table S16 (Supporting Information). h) Representative quantification of mean normalized fluorescence intensity variation registered during contraction of NMO‐rSkMs at day 35 of co‐culture not treated with cardiotoxin (no CTX) or 1 day, 5 days and 20 days after CTX treatment when stimulated with Glu. For each curve, data are shown as the mean of 3 independent replicates. i) Contraction quantification of NMO‐rSkM at D35 not treated with cardiotoxin (no CTX) or 1 day, 5 days and 20 days after CTX treatment. Contraction was recorded upon exogenous Glu administration, and is expressed as the maximum peak of fluorescence revealed via live imaging analysis. Quantifications were performed on three independent biological replicates per condition. Each of the three samples was divided into five ROIs and every dot represents a single ROI. Statistical significance was determined using Mann‐Whitney U test, ***p = 0.0002. Statistical results are reported in Table S17 (Supporting Information) j) Representative Z‐stack confocal immunofluorescence images of NMO‐rSkMs cross sections 20 days after CTX treatment, stained for TUJ1 (red), DESMIN (green) and α‐BTX^+^ regions (cyan). Scale bars, 50 µm (left) and 20 µm (right).

Following an acute injury, within a certain entity of damage, healthy SkM can spontaneously regenerate and rebuild functional tissue.^[^ ^35^, ^36^ ^]^ Based on the general tissue remodeling and the regeneration observed in NMO‐rSKM treated with CTX (Figure 4; Figure S8b, Supporting Information), we next investigated the functionality of assembloids before and after 1, 5, and 20 days from injury. To do so, we used two methods for quantifying the contraction upon both ACh and Glu stimulation (**Figure** **5**a–i). Specifically, we used a particle velocimetry imaging tool (PIVlab) to visualize and quantify the maximum displacement and the orientation of the displacement achieved by the entire samples before and after treatment with CTX using live imaging analysis. With the aim to first investigate the functionality of the muscular compartment, our assembloids were stimulated with ACh. As shown by the vector maps, and confirmed by quantification of the maximum displacement, we found that muscle contraction upon ACh stimulation was significantly reduced after 1 and 5 days from CTX treatment, and improved after 20 days from injury, when compared to untreated samples (Figure 5a,b). According to this data and to the morphometric parameters revealed above (Figure 4), the orientation of maximum muscle displacement follows preferential directions in untreated samples, while this directionality is almost lost after 1 and 5 days from injury, and ameliorates 20 days post‐damage (Figure 5c), suggesting the regeneration of oriented bundles of myotubes at the longer time point. Moreover, live imaging was also used to investigate the contraction and relaxation of the samples, which correspond to the fluorescence intensity variation registered upon neurotransmitter administration. In line with PIVlab results, the analysis performed on the fluorescence intensity variation during the time of selected region of interest showed an evident decrease in the contractile capacity of NMO‐rSKM assembloids after 1 and 5 days post‐CTX injury (107.41 ± 35.95 A.U. on day 1 and 278.41 ± 57.34 A.U. on day 5), that was rescued on day 20, when compared to assembloids analyzed before damage (1049.62 ± 157.87 A.U. on day 20 versus 832.65 ± 143.19 A.U. before damage; Figure 5d,e and Figure S8b (Supporting Information) and Video S4, Supplementary Video 4). Finally, samples were stimulated with Glu to assess the neuromuscular system regeneration. According to the dynamics of neuronal and muscular recovery observed in damaged assembloids (Figure 4), stimulation with Glu of NMO‐rSKM assembloids resulted in a reduced maximum displacement and not complete recovery of the NM system functionality after 20 days from CTX administration, when compared to untreated samples (18.11 ± 6.60 A.U. on day 1 versus 66.87 ± 8.36 A.U. on day 5 and 165.66 ± 24.81 A.U. on day 20; Figure 5f–i and Figure S8d (Supporting Information) and Video S5, Supplementary Video 5). In agreement, we found rare juxtapositions of pre‐and post‐synaptic elements of the NMJ 20 days after injury (Figure 5j). These data confirmed that NMO‐rSkM assembloids are able to respond to acute injury with SkM regeneration, and partial restoration of the NMJ functionality, suggesting a long‐lasting integration between NMO and rSkM.

## 3 Discussion

In this work, we developed a novel in vitro platform to investigate and reveal the complex cellular and biological events involved during the integration of the human NM system within an implantable tissue‐engineered muscle construct by producing a graft‐host SkM assembloid in vitro model.

Engineered SkM constructs hold the promise to be a therapeutic route for large and impactful muscle defects (single traumatic events, chronic conditions, and exercise‐induced injuries)^[^ ^43^, ^44^ ^]^ or congenital malformations (such as diaphragmatic hernia and abdominal wall defects).^[^ ^45^, ^46^ ^]^ However, their application in the clinic is still poor and it remains an issue of how to predict the efficiency of the developed engineered construct and establish the probability of success of engraftment and functional muscle regeneration in a humanized context. In addition, considering the application in the pediatric field for the treatment of congenital defects and malformations, it is extremely complicated to predict the response of a physiologically growing tissue/organ to the implantation of an engineered construct. Up to now, these aspects have been mainly investigated using various animal models.^[^ ^4^, ^47^, ^48^ ^]^ Animals offer a valuable opportunity to gain insight into regenerative mechanisms and systemic responses to the graft; however, ethical limitations coupled with species‐specific unpredictable biological responses, restrict their application in predicting the behavior of host human cells in relation to the graft.^[^ ^20^, ^21^ ^]^

Here we developed a humanized in vitro model where the muscular and neuronal contribution to the engineered SkM, and vice versa, could be studied in vitro. To do so, we combined hiPSC‐based organoid technology and a well‐established SkM tissue engineering approach to derive graft‐host SkM assembloids. To produce the assembloids, we first separately developed SkM‐engineered tissues and hiPSCs‐derived NMOs, and then we assembled those together to obtain graft‐host SkM assembloids. A precise modulation in terms of culture media in every single part of the process was required to sustain the growth and proliferation of both neuronal and muscular cell populations during culture.

In the last few years, the production of NMOs enabled the long‐term culture of a human NM system in vitro, allowing the investigation of interactions between MNs and SkM at the level of the NMJ in health and disease.^[^ ^22^, ^23^, ^24^, ^25^, ^49^ ^]^ Therefore, NMOs are generally considered a promising approach for modeling in vitro human NM biology, diseases, and muscle regeneration.^[^ ^22^, ^23^, ^24^, ^25^ ^]^ Based on this, we hypothesized that NMOs could be used as an archetype of transplant acceptance or failure of human origin to replace animal experiments, especially for mimicking fetal/neonatal NM systems that could mirror conditions of congenital malformations. We show that upon 22 days of hiPSCs differentiation, the derived NMOs presented cell types of the NM system, from cells that express early or late myogenic markers to the presence of neuronal protrusion toward the muscular compartment. One of the most relevant aspects for the positive outcome of a tissue‐engineered muscle graft is to understand whether the construct would integrate within the recipient tissue and vice versa, both from structural and functional points of view. In particular, in the SkM contest, the achievement of adequate and timely coordinated innervation by the host peripheral nervous system is mandatory to allow functional graft integration.^[^ ^18^ ^]^ Here, we show that tissue‐engineered products, such as dSkM and dSkM recellularized with hMPCs (i.e., rSkM), could be invaded, repopulated, and remodeled by muscular and neuronal cell types derived from NMOs, generating graft‐host SkM assembloids. These models allowed the in vitro evaluation of the interaction between the human NM system and engineered SkM constructs.

Until now, the most clinically relevant tissue engineering approach for volumetric SkM loss treatment has been performed using a decellularized urinary bladder scaffold.^[^ ^15^, ^16^ ^]^ Although treated patients recovered partial muscle volume and a certain degree of functionality, the study of Sicari and colleagues highlighted the importance of functional integration between the recipient tissue and the muscle graft for a positive outcome of the implant. We and other groups have demonstrated the significance of using tissue‐specific scaffolds to obtain relevant results in repairing muscle damages and congenital defects, being the dSkM intrinsically equipped with muscle‐specific and neurotrophic factors.^[^ ^5^, ^6^, ^14^ ^]^ Our study confirms this concept: we found that the presence of a muscle‐specific extracellular matrix allows cells to settle in anatomical positions closer to those observed in vivo (e.g., under the myofiber basal lamina for satellite cells). In addition, transplantation of the single decellularized scaffold for large muscle defects is not a guarantee of complete and long‐lasting treatment.^[^ ^13^, ^15^, ^16^ ^]^ Accordingly, we found that although the dSkM alone is able to attract muscle and neuronal cells from the NMO, the host‐cell invasion does not reach all areas of the scaffold in NMO‐dSkM assembloids. On the contrary, the presence of pre‐seeded hMPCs into dSkM (i.e., rSkMs) promotes a much more efficient and robust integration of cellular components from NMOs in NMO‐rSkM assembloids, suggesting that pre‐repopulation of decellularized scaffolds before implantation in a context of large muscle defects or volumetric muscle loss could greatly improve engraftment and functional restoration of the implant. Our observations were paralleled by significant improvement of the SkM contraction of the invaded tissue‐engineered construct, strongly suggesting that repopulating dSkM with tissue‐specific cells has a positive prediction to generate a functional graft once implanted in patients. The improved integration and functionality observed in NMO‐rSkM, when compared to NMO‐dSkM, might be due to paracrine signaling and small molecules secreted by hMPCs, both attracting muscle progenitor cells and axonal protrusions inside the scaffold and enhancing NM maturation of the assembloids. Moreover, pre‐seeding the decellularized scaffold with hMPCs has an effect on scaffold remodeling, which could be beneficial for NMO engraftment. Finally, the possible effect of myogenic pre‐seeded cells on the proliferation of NMO‐derived cells could be another potential mechanism associated with the higher levels of invasion when assembloids are produced in the presence rSkMs.

According to this, we also show that functional NMJs are generated upon the integration of the NMO‐derived neural compartment with rSkMs in NMO‐dSkM assembloids. Human NMJs have been shown to possess specific characteristics that distinguish them from comparable synapses in other mammalian species (e.g., mice and rats).^[^ ^50^ ^]^ These differences complicate the interpretation of animal studies for their applicability to humans. In our humanized in vitro 3D system, the presence of human NMJs formed by coin‐shaped patch endplates^[^ ^50^ ^]^ and capable of responding to neurotransmitters or toxins strongly suggest their similarity to NMJs present in vivo.

At the same time, the presence of stem and myogenic progenitor cells, such as PAX7^+^ cells identified in a satellite cell anatomical position, opened the possibility to investigate the long‐lasting ability of the integrated tissue‐engineered SkM construct upon damage. We can assume that PAX7^+^ cells are of the NMO origine since no PAX7^+^ cells could be found in rSkM. Thus, we used CTX, a well‐established in vitro and in vivo assay to induce a transient and reproducible acute injury of the myofibers without directly affecting the nerves.^[^ ^41^ ^]^ Our NMO‐rSkM assembloids responded in a synchronous and coordinated manner to CTX, with degeneration and subsequent regeneration of the muscular compartment, and delayed degeneration and partial regeneration of the neuronal compartment at comparable time points. This experimental setup confirmed the effective integration between NMO and rSkM and that NMO‐rSkM assembloids are able to undergo damage and respond with tissue regeneration, according to expected times and ways.^[^ ^42^ ^]^ Importantly, although SkM functionality was significantly rescued after 20 days from the injury (i.e., upon ACh stimulation), the regeneration of the neuronal compartment with functional NMJs was still not fully achieved in the same timeline (i.e., upon Glu stimulation). However, such regeneration capability of the neuronal compartment of assembloids could be also due to the action of neural progenitor cells identified in the NMO‐rSkMs. Based on this aspect and as mentioned before, we suggest that the developed platform holds particular translational relevance for studying tissue engineering strategies to treat congenital defects, such as diaphragmatic hernia and abdominal wall defects.^[^ ^45^, ^46^ ^]^ External stimulation such as electrical, mechanical, or other physical forces show promise in promoting in vivo and in vitro neural tissue regeneration.^[^ ^42^, ^51^, ^52^, ^53^ ^]^ After stimulation, neural tissue experiences increased proliferation, supporting axon sprouting and innervation. Thus, it could be of future interest to test whether external stimulations could accelerate NM system regeneration in our assembloids.

Despite the relevant results brought by our platform on the exploration of the interactions between the human NM system and engineered muscle tissue, it is important to underline that several key biological players are missing in the presented assembloid model. The future possibility to integrate into the NMO‐rSkM assembloids other cell types, such as those belonging to the vascular tissue and the immune system, remains a fascinating perspective that can open new possibilities to dissect the regulatory properties of other cellular players during the human NM system integration and regeneration.

In a broader scientific context in which researchers and clinicians increasingly seek to recreate 3D in vitro models able to mimic as much as possible the complex physiological environment of tissues or organs, our NMO‐rSkM assembloids can represent not only a preclinical test to predict the engraftment of specific muscle grafts but potentially also be a useful tool for studying drugs or therapeutic molecules for diseases that affect the NM system. The scientific community in the field of SkM and NM system research is strongly committed to the development of complex 3D in vitro models that can mimic myopathies and dystrophies, or the effect of aging on physiological mechanisms of repair and regeneration.^[^ ^54^, ^55^, ^56^, ^57^, ^58^, ^59^, ^60^ ^]^ In our 3D in vitro model, the presence of different cell types of the NM system can facilitate the understanding of the events that occur in muscular and NM diseases and regeneration.

Given its complexity in cellular composition, the high fidelity with the human context, and the demonstrated functionality even after acute damage, our assembloids can open new studies on regeneration mechanisms both in healthy and diseased contexts, for a better understanding of translational applications in SkM‐based regenerative medicine strategies.

## 4 Experimental Section

### Human‐Induced Pluripotent Stem Cell Derivation and Culture

All human iPSCs cell lines in this study were generated from foreskin fibroblast cell lines (BJ) by reprogramming in microfluidics.^[^ ^60^, ^61^ ^]^ We used BJ‐derived hiPSC lines wild‐type or constitutively expressing the GFP derived from the same hiPSC clone.

Human iPSCs were cultured in feeder‐free conditions on 0.5% Matrigel (MRF, Corning) coated cell culture plates (6‐multiwell, Sarstedt) in StemMACS iPS‐Brew XF (Miltenyi Biotec) with 1% penicillin/streptomycin (P/S; Gibco‐Fisher Scientific), at 37 °C in 5% CO_2_ in cell incubator. All cell lines were tested negative for mycoplasma and maintained below passage 30 before their use for differentiation. Human iPSCs at passage 12 were transduced with a lentiviral vector encoding for GFP and Puromycin resistance under the control of the PGK promoter. To enrich the human iPSC population for GFP‐positive cells, GFP‐transduced hiPSCs were both selected via puromycin treatment (1 µg mL^−1^ for 4 days) and sorted for GFP expression. For GFP‐hiPSCs sorting, cells were detached as single cells using TryplE Select (Gibco), resuspended in 1 mL PBS 1X, and sorted by flow cytometry. Sorted cells were resuspended in StemMACS iPS‐Brew XF (Miltenyi Biotec) with 1% penicillin/streptomycin (P/S; Gibco‐Fisher Scientific), supplemented with 10 µM StemMACS Y27632. Cells were then seeded in feeder‐free conditions onto 0.5% Matrigel (MRF, Corning) coated cell culture plates (6‐multiwell, Sarstedt).

### hiPSC differentiation for neuromuscular organoid derivation

For neuromuscular organoid (NMO) derivation, 15 µL of 100% MRF was dispensed on a sterile glass coverslip (Vetrotecnica) forming 1 cm^2^ of MRF droplet in 24‐multiwell plates (Sarstedt). MRF droplets were incubated at 37 °C for 25 min in the cell incubator to allow MRF polymerization. Before cell seeding (Day ‐2 of differentiation protocol), hiPSCs were enzymatically disassociated as single cells using TryplE Select (Gibco), 40 000 hiPSCs were resuspended in 50 µL of StemMACS iPS‐Brew XF (Miltenyi Biotec) supplemented with 10 µM StemMACS Y27632. Cells were seeded on top of Matrigel droplets and incubated for 90 min at 37 °C and 5% CO_2_ to allow cell adhesion prior to further media supplementation. After 500 µL media supplementation, cells were cultured for 24 h at 37 °C and 5% CO_2_ in the cell incubator. On Day ‐1 of the differentiation protocol, the media was changed, and cells were cultured in StemMACS iPS‐Brew XF. The differentiation protocol started at day 0 and was adapted from literature studies, taking advantage of small molecules and growth factors to direct neuromuscular differentiation.^[^ ^62^ ^]^ Briefly, from day 0 to day 2 the media was switched to a Dulbecco's Modified Eagle Medium/Nutrient Mixture F‐12 (DMEM F‐12, Gibco) based media, supplemented with Insulin‐Transferrin‐Selenium (ITS, Gibco), 1% P/S, 3 µM WNT agonist CHIRON99021 (Miltenyi Biotec) and 0.5 µM BMP antagonist LDN193189 (Miltenyi Biotec). From day 3 to 5, 20 ng mL^−1^ fibroblast growth factor‐2 (FGF‐2; Immunotools) were supplemented to the media. Starting from day 6, media was changed to DMEM F‐12, supplemented with 15% Knockout Serum Replacement (KSR; Gibco), 10 ng mL^−1^ hepatocyte growth factor (HGF; ImmunoTools), 2 ng mL^−1^ insulin‐like growth factor 1 (IGF‐1, Miltenyi Biotec), 20 ng mL^−1^ FGF‐2 and 0.5 µM LDN193189. From day 8 to day 11 of differentiation, cells were cultured in DMEM F‐12 supplemented with 15% KSR and 2 ng mL^−1^ IGF‐1. From day 12, the previous media was modified by including 10 ng mL^−1^ HGF and 2 ng mL^−1^ IGF‐1. From day 22, NMOs were cultured in myogenic secondary differentiation media (SDM) composed of DMEM F‐12 supplemented with 2% KSR, 1X ITS, 1% P/S, 1 µM CHIRON99021, 10 ng mL^−1^ ciliary neurotrophic factor (CNTF; PeproTech) and 10 ng ml^−1^ glial cell line‐derived neurotrophic factor (GDNF; PeproTech) until the endpoint of the experiment.^[^ ^29^ ^]^

### Human Adult Muscle Progenitor Cells (hMPCs) Isolation and Expansion

Primary human skeletal myoblasts were derived from healthy donor SkM biopsy. Patient tissues were collected after written informed consent following protocols number 2682P and number 3030P, approved by the Local Ethics Committee (Comitato etico per la sperimentazione clinica dell'Azienda Ospedaliera di Padova). From muscle biopsies, human muscle progenitor cells (hMPCs) were isolated using a previously established protocol.^[^ ^34^ ^]^

During cell expansion, hMPCs were cultured in proliferative medium (PM), composed of DMEM low glucose (1 g L^−1^ D‐glucose, Gibco‐Fisher Scientific), 20% fetal bovine serum (FBS; Gibco–Fisher Scientific) 10^−6^ M dexamethasone (Sigma‐Aldrich), 10 ng mL^−1^ bFGF (R&D System), 10 µg mL^−1^ insulin (Gibco‐Fisher Scientific) and 1% P/S.

To induce myogenic differentiation, confluent hMPCs were cultured in fusion medium (FM), composed of αMEM (Gibco‐Fisher Scientific) supplemented with 2% horse serum (HS; Gibco‐Fisher Scientific), 10 µg mL^−1^ insulin and 1% P/S.

### Flow Cytometry Analysis

Cell surface antigen expression was analyzed by flow cytometry for hMPCs characterization. Cells were detached with Trypsin‐EDTA treatment at different passages, up to passage 12. For surface antigen characterization, ≈1 × 10^4^ cells were incubated with anti‐CD56 PE antibody (BD Bioscience, Italy) and 7‐aminoactinomycinD (BD Bioscience, Italy), which was used to assess cell viability.

### Generation of a decellularized skeletal muscle tissue (dSkM)

Murine diaphragms were retrieved from 3 to 6‐months‐old C57BL/6j mice (protocol N. 1103/2016 and 418/2020‐PR approved by Animal Wellness local ethics committee, Organismo per il Benessere Animale OPBA, University of Padova and Fondazione Istituto di Ricerca Pediatrica Città della Speranza and Italian Ministry of Health). After collection, diaphragms were washed 2 times in 1X sterile phosphate‐buffered saline (PBS, Gibco‐Fisher Scientific) and then transferred in deionized water with 3% P/S, in order to start the decellularization process. Diaphragms were processed with three detergent‐enzymatic treatment (DET) cycles in order to achieve complete cell removal. Each DET cycle was composed of deionized water at 4 °C for 24 h, 4% sodium deoxycholate (Sigma‐Aldrich) at room temperature for 4 h, and 2000 Kunitz DNase‐I (Sigma‐Aldrich) in 1 M NaCl (Sigma‐Aldrich) at room temperature for 3 h.^[^ ^32^ ^]^ After decellularization, decellularized SkM (dSkM) were washed for at least 6 days in PBS 1X and immediately used or preserved in liquid nitrogen.

### dSkM Recellularization Protocol

Mouse dSkM was used for the recellularization protocol with hMPCs. Each decellularized diaphragm was divided into four parts, each measuring ≈30 mm^2^, excluding the tendon and the crural muscle. Each part was fixed with four stainless steel pins on polydimethylsiloxane support (PDMS 9:1; Sylgard 184, Dow Corning), and placed into a well of a 24‐well plate. HMPCs, previously expanded for 7–12 passages, were resuspended in 40 µL of 13% Collagen type I (Sigma‐Aldrich), 10% Fibronectin (Sigma‐Aldrich), 10% IGF‐1 (ImmunoTools) in PM. To produce recellularized SkM (rSkM) the mix was injected inside the dSkM at a density of 1 × 10^6^ cells per 30 mm^2^, and incubated for 3 h at 37 °C and 5% CO_2_, prior to further media supplementation. 3 h from the injection, 500 µL of PM were added to each well. PM was changed every 48 h, and on day 4 it was replaced with FM. On day 5, media were exchanged again, with a mix composed of 50% FM and 50% SDM.

### Seeding of NMOs on dSkM or rSkM and assembloid generation

D22 NMOs were seeded alternatively on dSkMs or on rSkMs (recellularized for 6 days). To facilitate NMO adhesion to the scaffolds, media was removed from the scaffolds and D22 NMOs from in suspension culture were carefully placed above the rSkM, and covered with 5 µL 100% MRF. The constructs were located at 37 °C in the cell incubator for 5 min to allow MRF polymerization. After MRF polymerization, 500 µL SDM were added to each well. Samples were maintained in SDM until the established experimental endpoint, media were changed every 72 h.

### Live Imaging and Contraction Analysis

At the defined ending time point, all live imaging analyses were performed using Leica M205 FCA stereomicroscope equipped with PLANAP0 1.0× objective, with an acquisition rate of 5 to 10 frames per second. When indicated, samples were stimulated with glutamate (Glu) or acetylcholine (ACh) neurotransmitters. The Glu solution was prepared using L‐Glutamic acid powder (Sigma‐Aldrich) dissolved in sterile water to obtain a 100 mM stock solution. ACh (Sigma‐Aldrich) was reconstituted in PBS to produce a 100 mM stock solution. Each neurotransmitter solution was administered to samples during live imaging acquisition at the final working concentration of 10 µM. For bungarotoxin (BTX) treatment, samples were incubated with 1 µg mL^−1^ α‐bungarotoxin Alexa FluorTM 555 (Invitrogen, B13422) for 12 h at 37 °C prior to live imaging acquisition and analysis.

For spontaneous contraction analysis, samples were monitored over time using bright field or fluorescence imaging. Pixel intensity variation during live imaging acquisition was measured considering whole mount samples or, where indicated, considering specific regions of interest (ROIs), accurately selected in correspondence with myogenic areas. Quantifications were performed on three independent biological replicates per condition, each sample was divided into five ROIs and each dot represents a single ROI. Pixel intensity variation was quantified using alternatively the Quantify tool of the Leica Application Suite X (LAS X) software or ImageJ Plugin Spiky.^[^ ^63^ ^]^

Both LAS X and Spiky were used to extract intensity signals from live recordings, which were exported as .csv files and then used for further imaging analyses. These csv files containing raw intensity variation data were processed by implementing a MATLAB 2021 script (MathWorks) with the goal of correcting photobleaching and to be able to remove eventual neurotransmitter administration‐based artifacts.

To remove this artifact, a MATLAB internal code was used. At first, we proceeded with the manual selection of the artifact's start and conclusion points through a MATLAB dialog box interface. Subsequently, the code eliminates the undesirable signal segment, to then apply the Modified Akima piecewise cubic Hermite interpolation technique, present by default in MATLAB. This interpolation performs cubic interpolation to produce piecewise polynomials with continuous first‐order derivatives to avoid excessive local undulations. The distinction between this and the standard Akima algorithm lies in its prioritization of slopes when two different ones intersect; the Modified version favors the slope closer to the horizontal direction in order to prevent overshooting.^[^ ^64^, ^65^ ^]^

For vector map generation and displacement measurements, a graphical user interface (GUI) based particle image velocimetry software (PIVlab) version 3.07 was used according to the literature.^[^ ^61^, ^62^, ^63^ ^]^ Briefly, selected pairs of frames acquired during live imaging analysis of wholemount samples were cross‐correlated to yield vectorial maps and local displacement. In particular, the maximum displacement was quantified by cross‐correlating pairs of frames acquired before contraction and at the maximum displacement of samples. Quantification of the displacement and direction of vectors was obtained using the Multipass Fast Fourier Transform (FFT) window deformation PIV algorithm and stored as a correlation matrix in a .mat file. Polar charts showing the relation between displacement and direction of vectors for each sample were built from a custom‐made R (v. 4.3.1) script using package ggplot2 v. 3.5.1.^[^ ^64^ ^]^ All mentioned MATLAB algorithms have source code available in MATLAB documentation. All scripts and custom code are available upon request to the authors.

### Calcium Transient Analysis

For calcium transient evaluation, samples at the established experimental endpoint were incubated with Fluo‐4‐AM (Thermo Fisher Scientific F14201) following the manufacturer's instruction. To avoid GFP interference with Fluo‐4 evaluation, we included hiPSC that did not express GFP in this set of experiments. Briefly, media was removed and samples were incubated with 20 µM Fluo‐4‐AM, 5 µL mL^−1^ Pluronic F‐127 (Thermo Fisher Scientific), and 12.5 µL mL^−1^ sulfinpyrazone (Sigma‐Aldrich) in serum‐free cell medium for 30 min at 37 °C and 5% CO_2_. Samples were then accurately washed with myogenic SDM without KSR and subjected to live imaging analysis. Live imaging was performed as previously described in whole‐mount samples.

For calcium transient analysis, ≥ 10 ROIs per sample with a diameter of 20 µm were selected to evaluate the mean of fluorescence intensity variation of a restricted group of myofibers, which were identified thanks to their morphology. ROI fluorescence intensity variations were analyzed using the tool Process of the LAS X software or ImageJ plugin Spiky. Obtained data were processed and normalized with the same MATLAB code described for contraction to express calcium variation during the time of imaging acquisition.

For calcium ΔF/F_0_ calculation, the same selected ROIs used for calcium flux analysis were employed to identify baseline (before neurotransmitter stimulation, F_0_) and maximum (after neurotransmitter stimulation, F_peak_) fluorescence intensity values. Automatic calcium ΔF/F_0_ calculation requires, as an input, .csv intensity signals extracted from a live recording, either through the Spiky plugin or directly via the LAS X. These signals need to be near the event of interest and have a vector size of at least 50. A tailored MATLAB code was designed to process these intensity signals, automatically determining the baseline as the mean of the first 20 points using the mean function of MATLAB, and Fpeak as the maximum signal value for each ROI *max* function of MATLAB. The resulting data was then printed in matrix format, to be easily exported for further analysis and comparison in Excel.

All mentioned MATLAB algorithms have source code available in MATLAB documentation. All ImageJ plugins are licensed under open‐source GNU GPL v.3 license.

### Cardiotoxin Injury

For cardiotoxin (CTX, Latoxan, Portes‐lés‐Valence, France) injury, NMO‐rSkM samples were treated with CTX at a final concentration of 0.2 µM.^[^ ^31^ ^]^ 35 days after NMO seeding on rSkM, CTX was added directly to the secondary differentiation media and was maintained for 6 h. Samples were then maintened in cultured without CTX and analyzed at different timepoints.

### Histology

NMO‐dSkM and NMO‐rSkM were fixed for 1 h in 4% paraformaldehyde (PFA, Sigma‐Aldrich) at room temperature. Histology was evaluated on frozen sections (15 µm thick) stained with hematoxylin and eosin kit for rapidly frozen sections (Bio‐Optica, Milan, Italy), following the manufacturer's instructions.

### Immunofluorescence Analyses

NMO‐dSkM and NMO‐rSkM were fixed for 1 h at 4% PFA at room temperature. Samples were then washed in PBS and analyzed in whole mount or in 30 µm cross‐sections. For cryosection staining, samples were embedded in an optimum cutting temperature (OCT) compound (Sakura 4583) and sectioned using CM1950 cryostat (Leica). Samples were washed with PBS for 5 min and blocked at room temperature in 1% BSA, 0.5% Triton X‐100 (Sigma) in PBS solution (PBST) for 2 h. Primary antibodies (Table S18, Supporting Information) were diluted in 1% BSA PBST solution and incubated for 48 h at 4 °C for whole‐mount staining, and overnight at 4 °C for cryosections. Samples were then washed at least three times for 30 min each in 1X PBST solution on an agitation plate for whole mount staining, and at least three times for 15 min each in PBS solution for cryosections. Samples were then incubated for 48 h (whole mount) at 4 °C or 2 h at room temperature (cryosections) with secondary antibodies solution (Table S19, Supporting Information). After incubation with secondary antibodies, samples were washed twice with PBS for 30 min each on an agitation plate (whole mount) or for 10 min (cryosections). For whole‐mount staining, nuclei were counterstained with 10 µg mL^−1^ Hoechst 33 342 (Thermo Fisher). For cryosections, nuclei were counterstained alternatively with fluorescent mounting medium plus 100 ng mL^−1^ 4′,6‐diamidino‐2‐phenylindole (DAPI, Sigma‐Aldrich) or with 10 µg mL^−1^ Hoechst 33 342 (Thermo Fisher).

Images were acquired using an LSM800 inverted confocal microscope (Zeiss) and Thunder fluorescent stereomicroscope (Leica M205 FCA) equipped with PLANAP0 1.0X objective.

### Immunofluorescence Quantification

We used Fiji ImageJ software^[^ ^66^ ^]^ for adjustments of brightness and contrast levels, to merge image channels, to obtain standard deviation intensity projections and 3D image reconstructions, and to measure sample areas, myotube, and neural projection length and thickness. For quantification purposes, 6 to 10 immunofluorescence images of three independent biological replicates for each sample were converted into 8‐bit images and then analyzed with the measure function of ImageJ software.

Areas were measured on calibrated images using the ImageJ drawing tool to outline sample areas and then using the measure tool to quantify areas.

To quantify the number of PAX7^+/−^, PAX7^+^Ki67^+/−^ and PAX7^+^ located under or out of basal lamina the Cell Counter tool of ImageJ software (Fiji, v.2.14.0/1.54f) was used. The number of positive cells for each staining was normalized to the total number of PAX7^+^, except for PAX7^+/−^ cells which were normalized on the total cells in the field, and expressed as a percentage.

Axonal projection length was quantified using the ImageJ plugin NeuronJ.^[^ ^65^ ^]^

For fluorescence intensity quantifications, we converted images into 8‐bit, and then, using the measure tool, we quantified area integrated density and mean grey value. Corrected total cell fluorescence (CTCF) was normalized for background by subtracting the integrated density of each selected ROI from the background signal, using the following equation: CTCF = Integrated Density – (Area of selected cell X Mean fluorescence of background readings).^[^ ^66^ ^]^

Comparable‐sized ROIs were chosen for each of the analyzed images. All mentioned ImageJ plugins have source code available and are licensed under open‐source GNU GPL v.3 license.

### RNA Purification and RT‐qPCR

Total cell RNA was isolated and purified using RNeasy Plus Mini Kit (Qiagen) according to the manufacturer's instructions. Samples were accurately disrupted and homogenized with sterile scissors prior to extraction. All the harvested and homogenized samples were lysed with RNeasy Plus lysis buffer (RLT, Qiagen) and further processed according to the protocol. Extracted RNA quality and concentration were assessed using Nanodrop (Thermo Scientific). Complementary DNA (cDNA) was obtained from samples using a High Capacity cDNA Reverse Transcription Kit (Applied Biosystems), in a dedicated thermocycler (Mastercycler X50a, Eppendorf).

Expression of myogenic and neural markers was quantified by using a 7500 Fast Real‐Time PCR System (Applied Biosystem) and Platinum SYBR Green SuperMix kit components (Invitrogen, 11733‐038) according to the manufacturer's instructions. All the primers used are listed in Table S20 (Supporting Information).

Target Ct values of gene expression were normalized to that of GAPDH or Β2‐microglobulin, used as housekeeping genes. Data are shown as relative fold change in gene expression applying the 2^−ΔΔCT^ method.

### Statistical Analyses

All analyses were performed with GraphPad Prism 9. Data are expressed as mean ± SEM or mean ± s.d. of multiple biological replicates (as indicated in figure legends).

Statistical significance was determined using the Mann‐Whitney U test. A *p*‐value below 0.05 was considered statistically significant. Data supporting the findings of this study are available from the corresponding author upon reasonable request.

## Conflict of Interest

The authors declare no conflict of interest.

## Author Contributions

A.U. and M.P. designed the study. L.R. performed the co‐seeding experiments and analyses and B.A. helped. B.A. produced NMOs and performed molecular analysis and L.R. helped. L.R. and E.C. processed muscle biopsies, and derived and characterized the hMPCs. L.R. and M.L.P. performed the live imaging analyses of fluorescence intensity variation. L.S. performed PIVlab analysis and graphical representation and B.A. helped. G.C. and P.C. contributed to sample preparation and imaging analysis. L.R. and E.M. derived dSkMs. O.G. and S.A. produced and characterized the wild‐type hiPSC line. C.La. produced and characterized the GFP hiPSCs. N.E. supervised the hiPSCs production and contributed to data interpretation. A.U. coordinated and supervised the NMO and assembloid production and use. All the authors contributed to the revision of the manuscript. A.U. and M.P. analyzed and interpreted the data, wrote the manuscript, and supervised the project. L.R. and B.A. contributed equally.

## Supporting information



Supporting Information

Supplemental Video 1

Supplemental Video 2

Supplemental Video 3

Supplemental Video 4

Supplemental Video 5

## Data Availability

The data that support the findings of this study are available from the corresponding author upon reasonable request.
