# Supplementary material for: Engineering Assembloids to Mimic Graft‐Host Skeletal Muscle Interaction
Source: Adv Healthc Mater. 2025 May 5;14(17):2404111. doi: 10.1002/adhm.202404111 (PMC12232128; doi:10.1002/adhm.202404111)
Supplement: Supplementary file 1 — Supporting Information [file ADHM-14-0-s002.docx]

**Supplementary material for:**

**Engineering assembloids to mimic graft-host skeletal muscle interaction**

**Authors:**

Lucia Rossi^1,2^*, Beatrice Auletta^1,2,3^*, Luigi Sartore^1,2^, Marco La Placa^2^, Giada Cecconi^1,2^, Pietro Chiolerio^1,2^, Edoardo Maghin^4,5^, Silvia Angiolillo^3,6^, Eugenia Carraro^4^, Onelia Gagliano^3,6^, Cecilia Laterza^6,7^, Nicola Elvassore^3,6^, Martina Piccoli^4*^ and Anna Urciuolo^1,2*^

^1^ Department of Molecular Medicine, University of Padova, Via G. Colombo 3, 35131 Padova, Italy

^2^ Neuromuscular Engineering lab, Istituto di Ricerca Pediatrica, Città della Speranza, Corso Stati Uniti 4/F, 35127, Padova, Italy

^3^ Department of Industrial Engineering, University of Padova, Via Gradenigo 6/a, Padova, 35131, Italy

^4^ Tissue Engineering lab, Istituto di Ricerca Pediatrica, Città della Speranza, Corso Stati Uniti 4/F, 35127, Padova, Italy

^5^ Department of Chemical and Pharmaceutical Sciences, University of Trieste, Italy

^6^ Veneto Institute of Molecular Medicine, Via Orus 2, Padova, 35131, Italy.

^7^ Department of Biomedical Sciences, University of Padova, via Ugo Bassi 58/B, 35131, Padova, Italy

Co-correspondence to:

Anna Urciuolo, [anna.urciuolo@unipd.it](mailto:anna.urciuolo@unipd.it)

Martina Piccoli, [m.piccoli@irpcds.org](mailto:m.piccoli@irpcds.org)

*These authors contributed equally

**This file includes:**

**Supplementary Figures 1-8, Supplementary Tables 1-20 and captions of Supplementary Videos 1-5.**

**Supplementary Figures**


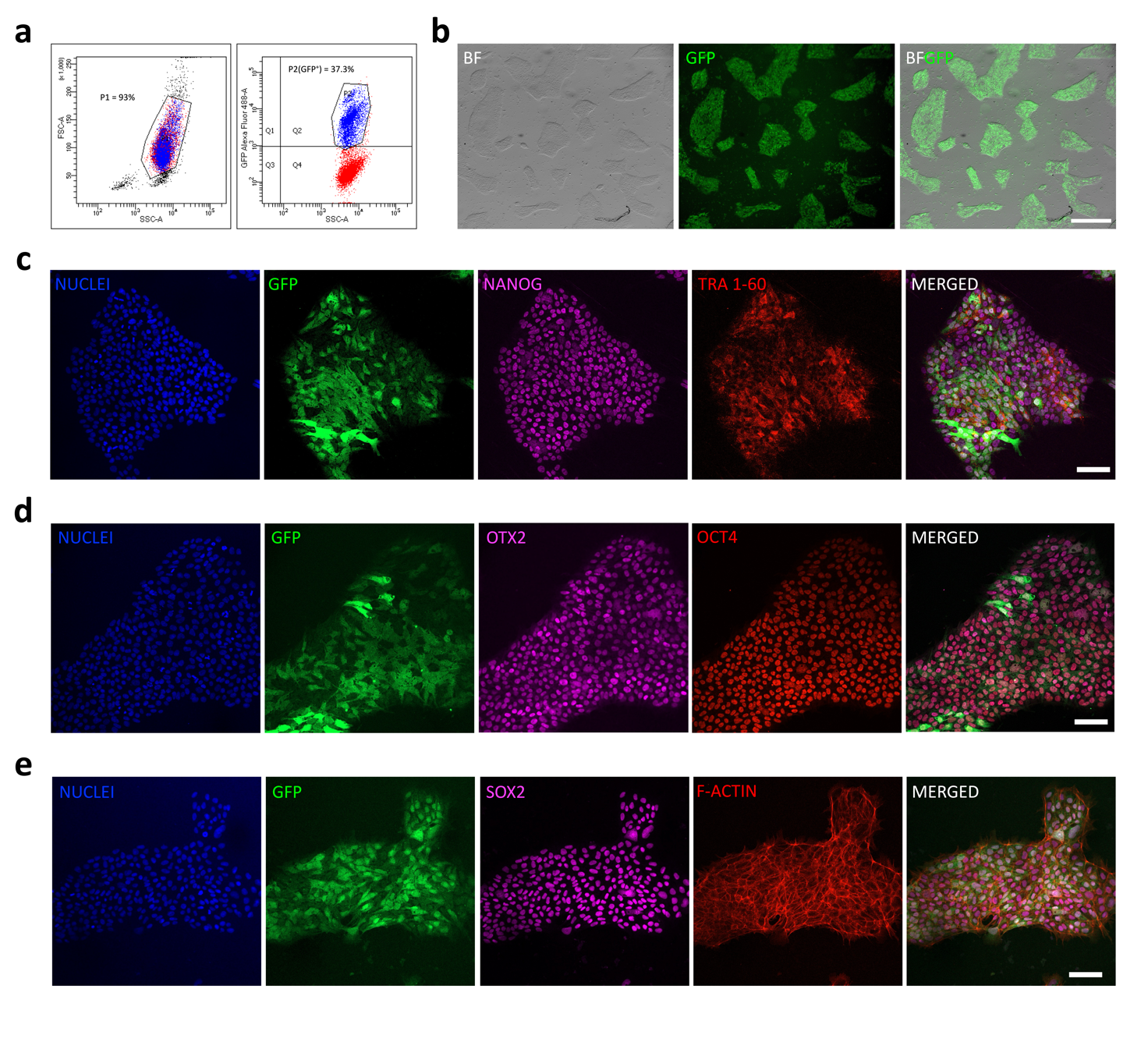


**Supplementary Figure 1. GFP-hiPSC characterization. a**. Flow cytometry plots showing the gating of the alive cells selected based on FFS and SSC (left) and the sorted cells based on GFP positivity (blue dots) representing 37,3% of the total cells. **b**. bright field and fluorescence images of the iPSCs after one passage from sorting. **c-e**. Immunofluorescence staining for pluripotency-related markers (i.e. NANOG (purple), TRA1-60 (red) in panel c; OTX2 (purple), OCT4 (red) in panel d; SOX2 (purple) and F-Actin (red) in e). Nuclei are counterstained with DAPI (blue). Scale bars are 500um in panel b and 100um in panels c, d, e.


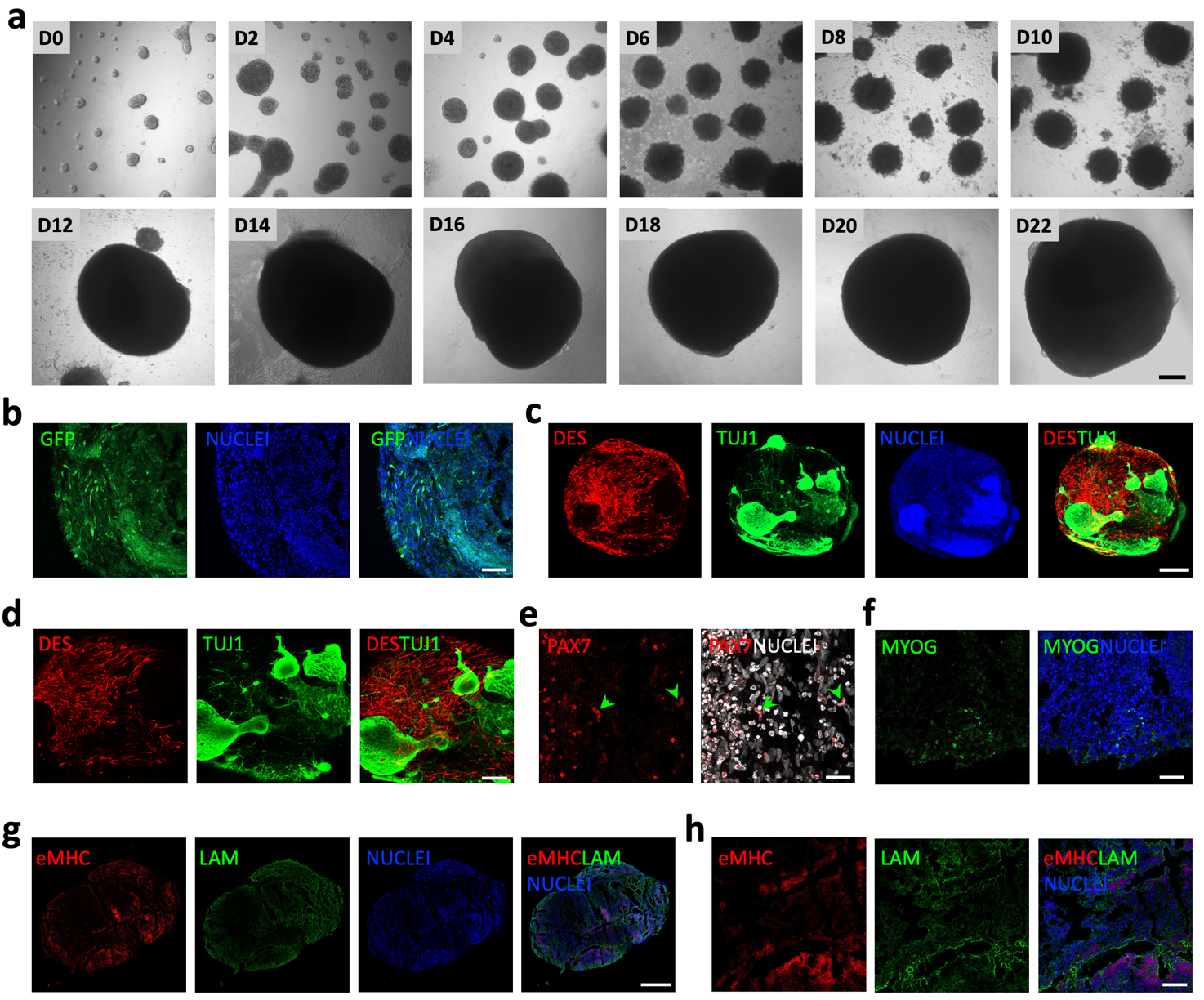
**Supplementary Figure 2. Neuromuscular organoids (NMOs) evolution over time and NMOs characterization on day 22. a**. Representative brightfield images showing the evolution of NMOs from day 0 (D0) to day 22 (D22). Scale bar, 200 µm. **b**. Representative Z-stack confocal immunofluorescence images of NMO cross-sections stained for GFP (green). Nuclei are counterstained with Hoechst (blue). Scale bar, 100 µm. **c-d**. Representative Z-stack confocal immunofluorescence images of NMO stained in whole mount for DESMIN (red) and TUJ1 (green). Nuclei are counterstained with Hoechst (blue). Scale bars, 200 µm (panel c) and 100 µm (panel d). **e**. Representative Z-stack confocal immunofluorescence images of NMO cross-sections stained for PAX7 (red). Nuclei are counterstained with Hoechst (grey). Green arrowheads indicate PAX7^+^ nuclei. Scale bar, 20 µm. **f**. Representative Z-stack confocal immunofluorescence image of NMO cross-sections stained MYOG (green). Nuclei are counterstained with Hoechst (blue). Scale bar, 50 µm. **g-h**. Representative Z-stack confocal immunofluorescence images of NMO cross-sections stained for embryonic MHC (red) and LAMININ (green). Nuclei are counterstained with Hoechst (blue). Scale bars, 500 µm (panel g) and 100 µm (panel h).


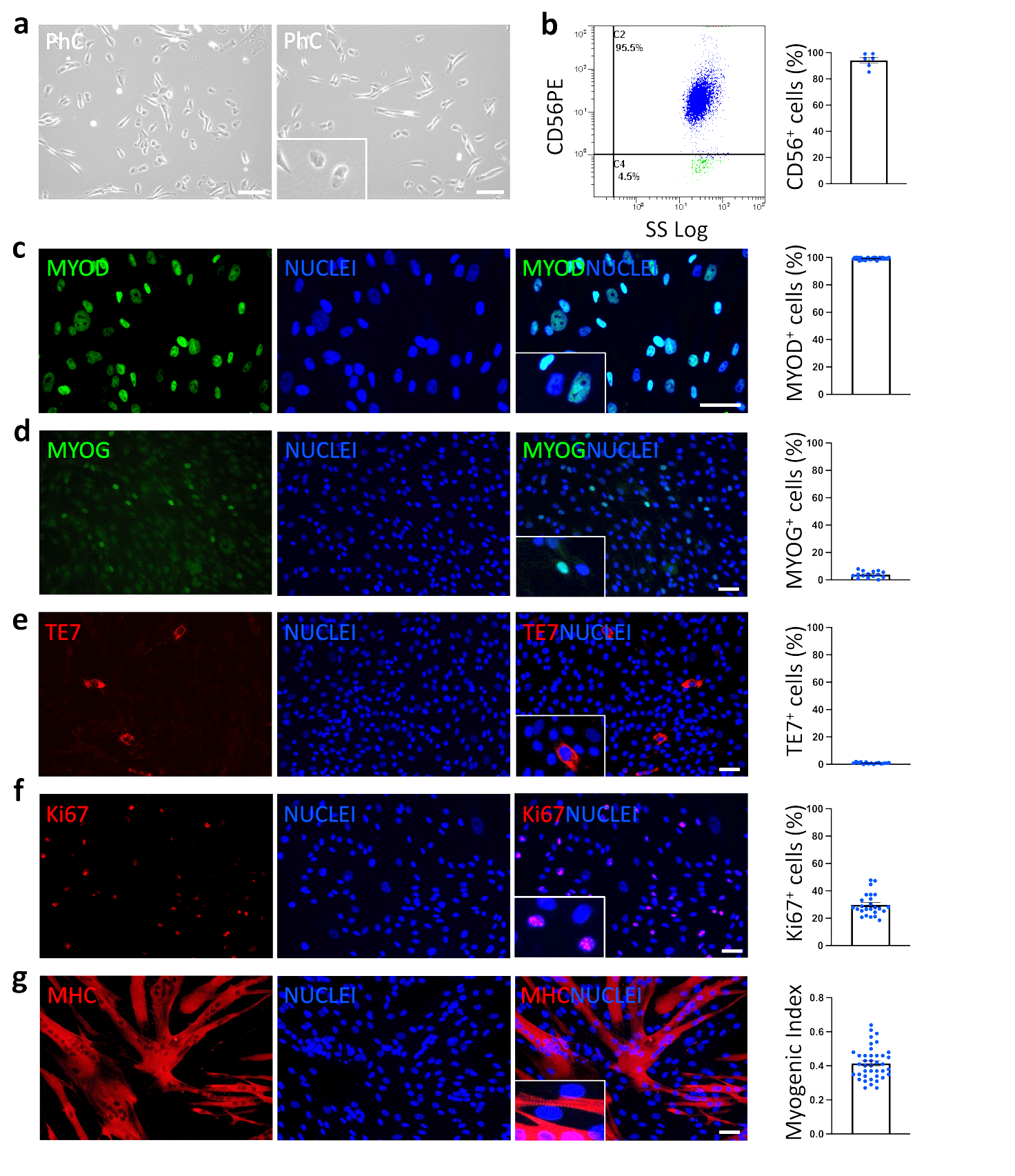


**Supplementary Figure 3. Human Muscle Precursor Cells (hMPCs) characterization in 2D culture. a**. Representative phase contrast (PhC) images showing hMPCs cultured in vitro. Scale bar, 100 µm. **b**. Left: Flow cytometry plot showing cell positivity for CD56 surface cell marker. Flow cytometry was repeated up to passage 12 (p12). Right: histogram showing mean ± SEM of n=5 flow cytometry analyses, representing CD56+ cells, expressed in percentage. **c-g**. Representative immunofluorescence images and quantifications of hMPCs cultured in vitro in proliferating (c-f) or differentiating (g) conditions and stained for MYOD (green), MYOG (green), TE7 (red), Ki67 (red), and MHC (red). Nuclei are counterstained with Hoechst (blue). Quantification of hMPCs myogenic index at p8. Scale bars, 50 µm.


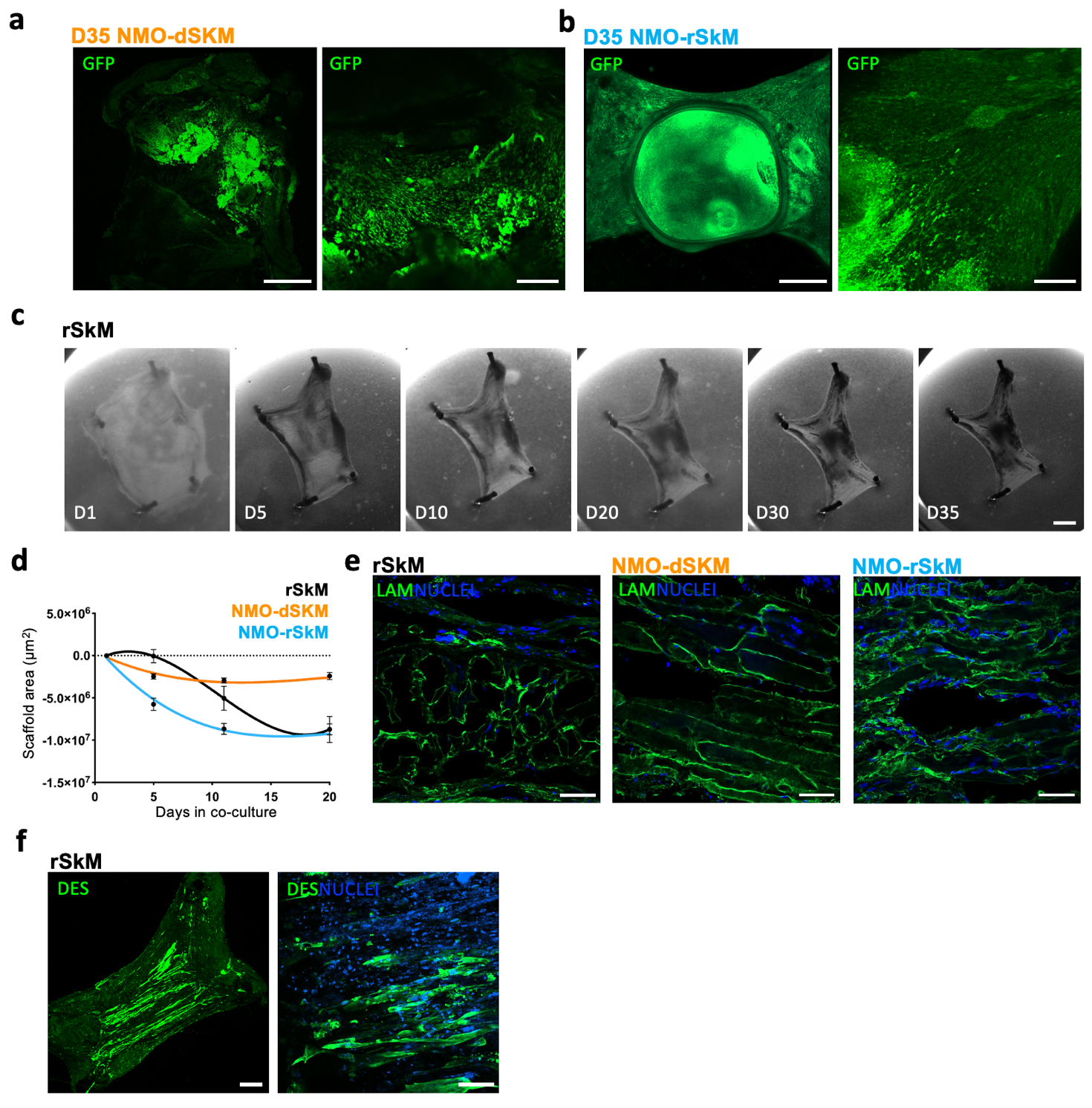


**Supplementary Figure 4. Comparison between rSkM, NMO-dSkM and NMO-rSkM relative to Figure 2b-d and Figure 2. a**. Representative confocal images showing GFP+ cells invading the scaffold in D35 NMO-dSkM. Scale bars, 1 mm (left) and 200 µm (right). **b**. Representative confocal images showing GFP+ cells invading the scaffold in D35 NMO-rSkM. Scale bars, 1 mm (left) and 200 µm (right). **c**. Representative brightfield images showing the evolution of rSkMs from day 1 (D0) to day 35 (D22). Scale bar, 1 mm. **d**. Scaffold area variation (µm^2^) over culture time (days), expressed as difference between scaffold area at day n and scaffold area at day 1 of co-culture. Data are shown as mean ± SEM of n>9 independent replicates. **e**. Representative Z-stack confocal immunofluorescence images of rSkM (D40), NMO-dSkM (D35) and NMO-rSkM (D35) cross-sections stained for LAMININ (green). Nuclei are counterstained with Hoechst (blue). Scale bars, 50 μm. **f**. Representative Z-stack confocal immunofluorescence images of whole mount rSkM after 40 days in culture, stained for DESMIN (green). Nuclei are counterstained with Hoechst (blue). Nuclei are counterstained with Hoechst (blue). Scale bars, 500 μm (left), 200 μm (right).


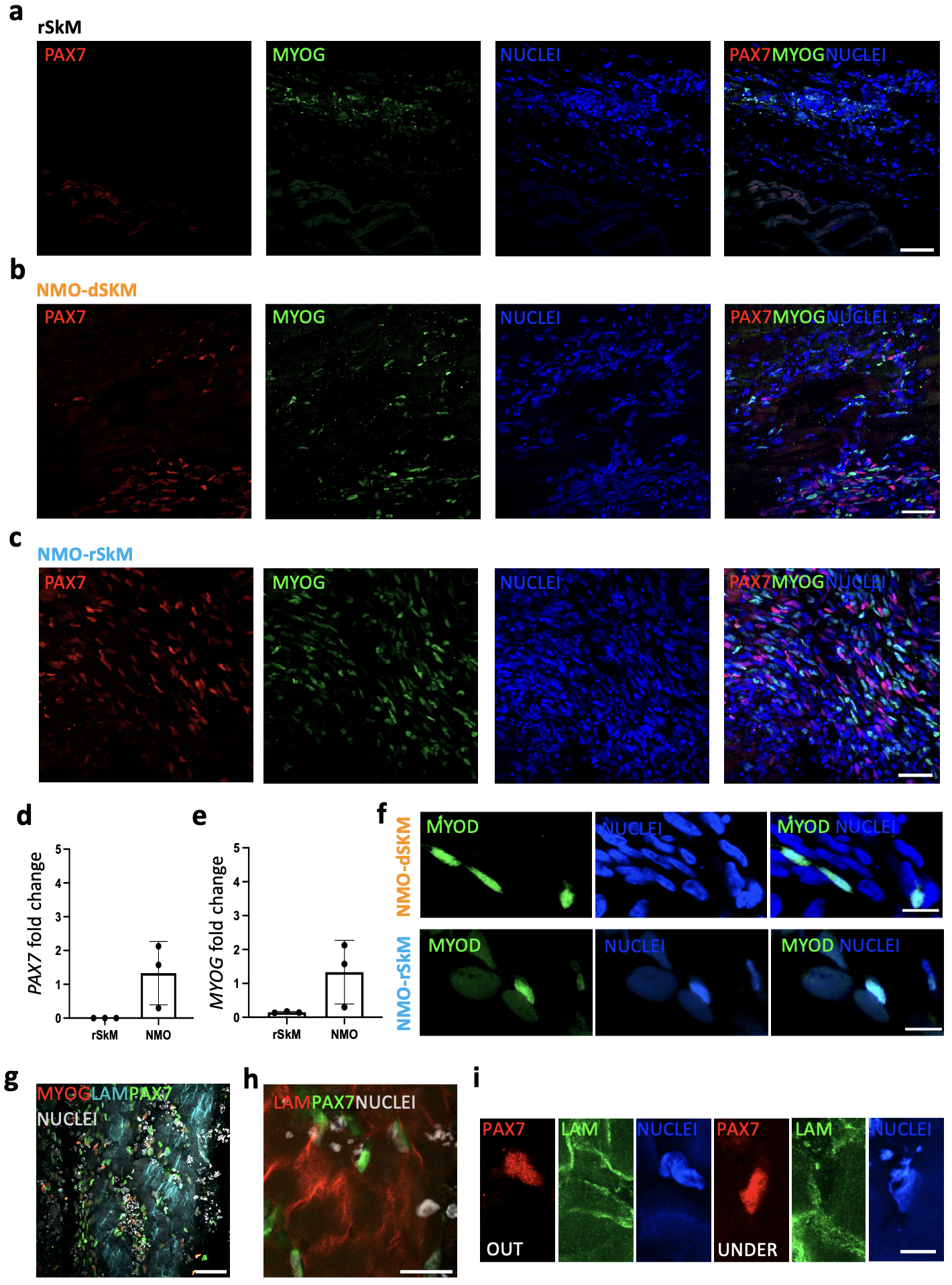


**Supplementary Figure 5. Myogenic stem and committed cell characterization. a-c** Representative confocal immunofluorescence image of rSkM (a, D40), NMO-dSkM (b, D35) and NMO-rSkM (c, D35) cross-sections, stained for PAX7 (red) and MYOG (green). Nuclei are counterstained with Hoechst (blue). Scale bar, 50 µm. **d**. *PAX7* gene expression in rSkMs and in NMOs. Data normalized to housekeeping *B2-microglobulin* gene expression and shown as fold change over NMO. Data are shown as mean ± s.d. of 3 independent biological replicates. **e**. *MYOG* gene expression in rSkMs and in NMOs. Data normalized to housekeeping *B2-microglobulin* gene expression and shown as fold change over NMO. Data are shown as mean ± s.d. of 3 independent biological replicates. **f**. Representative confocal immunofluorescence image of D35 NMO-dSkM and NMO-rSkM cross-sections, stained for MYOD (green). Nuclei are counterstained with Hoechst (blue). Scale bar, 10 µm. **g.** Representative Z-stack confocal immunofluorescence image of NMO-rSkM cross-sections at day 35 of co-culture stained for MYOG (red), LAMININ (cyan) and PAX7 (green). Nuclei are counterstained with Hoechst (grey). Scale bar, 50 µm. **h.** Representative Z-stack confocal immunofluorescence image of NMO-rSkM cross-sections at day 35 of co-culture stained for LAMININ (red) and PAX7 (green). Nuclei are counterstained with Hoechst (grey). Scale bar, 20 µm. **i.** Representative confocal immunofluorescence image NMO-rSkM cross-sections at day 35 of co-culture showing different localization of PAX7 (red) out (left panel) or under (right panel) basal lamina (LAM, green). Scale bars, 10 µm.


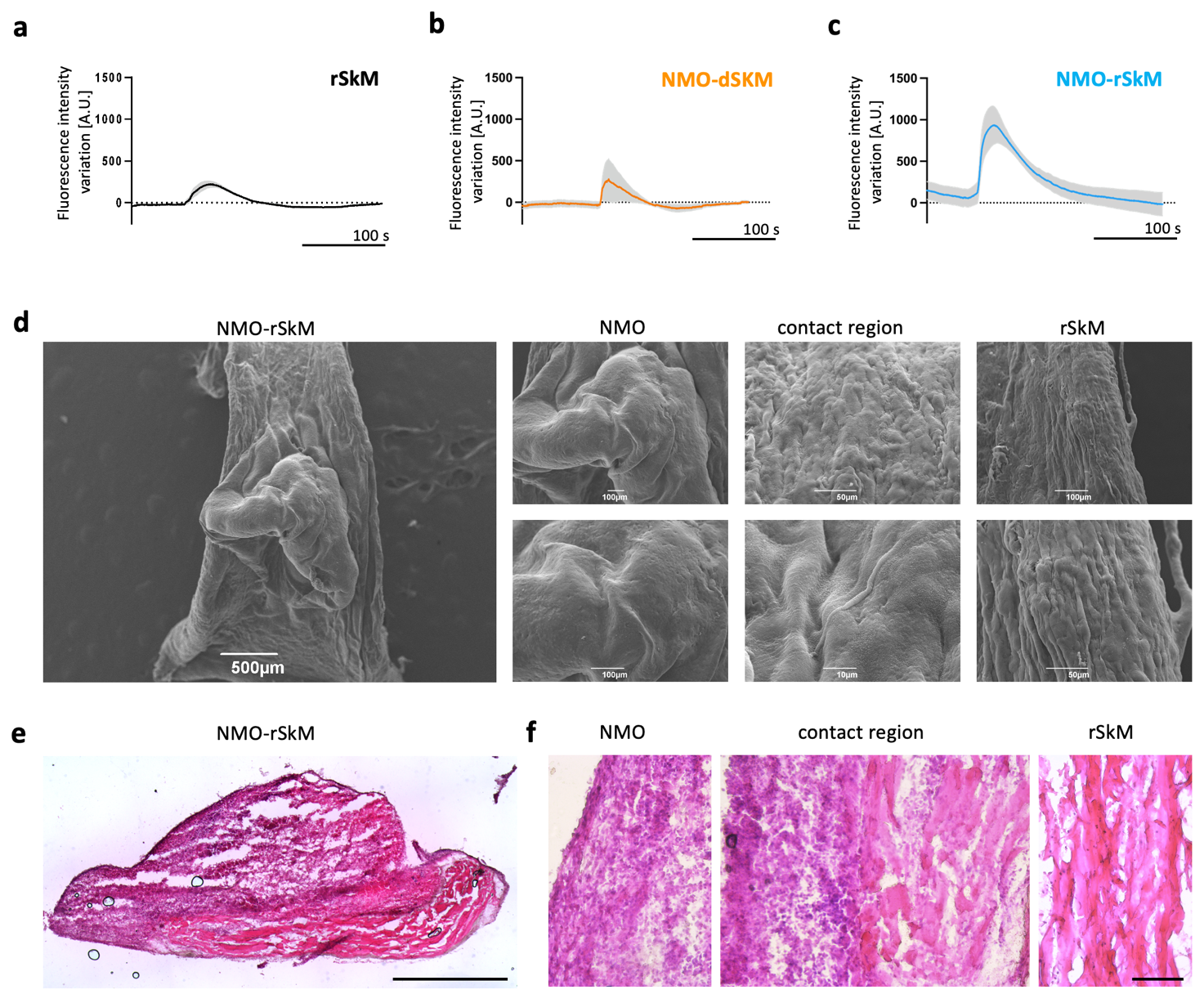


**Supplementary Figure 6.** **Functional and morphometric characterization of assembloids. a-c**. Representative quantification of mean normalized fluorescence intensity variation registered during live imaging analysis of rSkM, NMO-dSkMs, and NMO-rSkMs, stimulated with ACh. Data are shown as mean ± SEM of 9 ROIs, 3 ROIs were selected from each of 3 independent replicates. The dotted line corresponds to the baseline equal to 0. **d**. Representative scanning electron microscopy images showing a panoramic image of NMO-rSkM sample and magnifications for each region of interest (NMO, rSkM and region of contact between the two). Scale bars indicated within images. **e.** Hematoxylin and eosin staining on D35 NMO-rSkM cross section. Scale bar, 1 mm. **f**. Zoom of hematoxylin and eosin staining on D35 NMO-rSkM cross section. Scale bar, 100 μm.

**
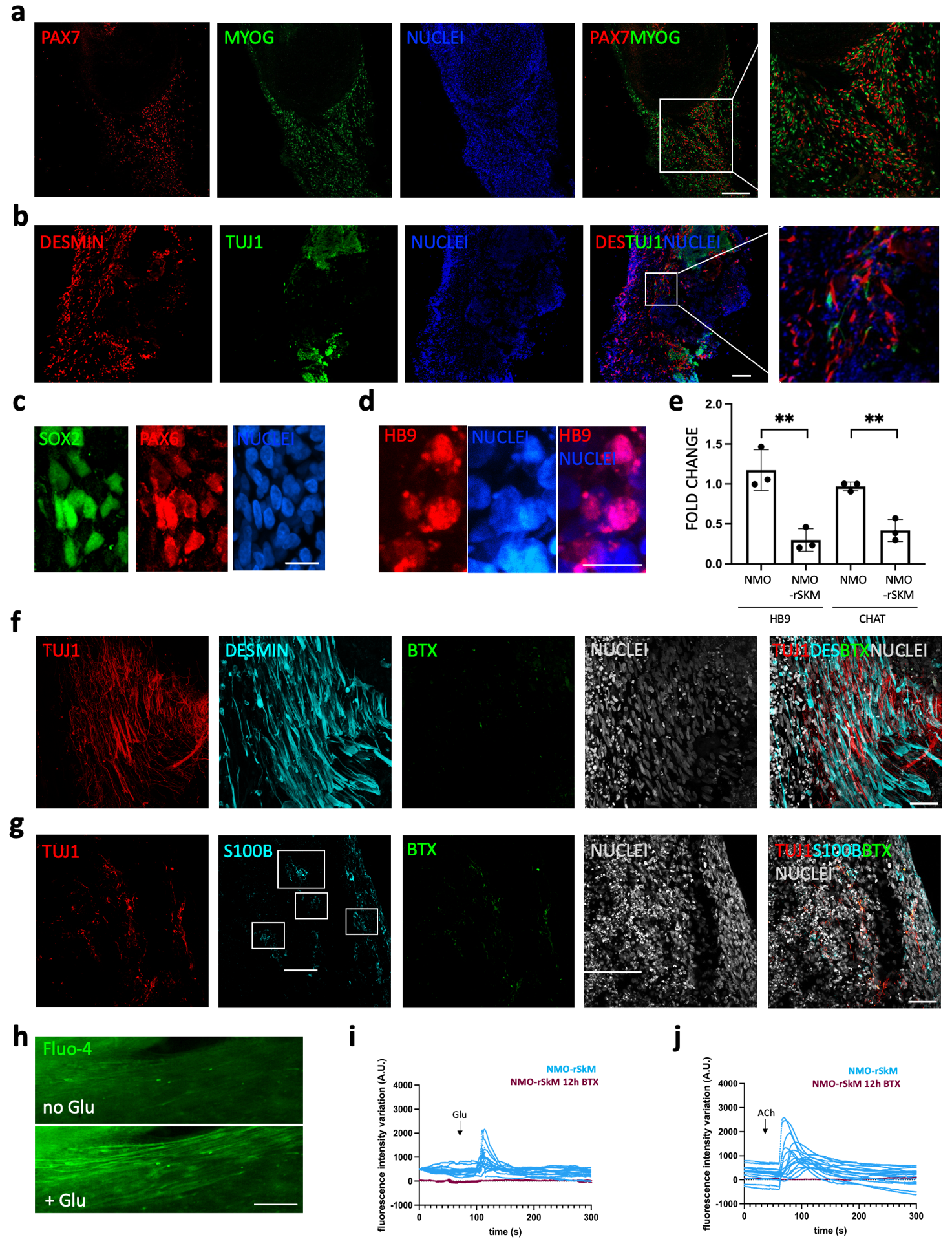
**

**Supplementary Figure 7. a**. Representative Z-stack confocal immunofluorescence image of NMO-rSkM cross-sections stained for PAX7 (red) and MYOG (green). Nuclei are counterstained with Hoechst (blue). Scale bar, 200 µm. **b**. Representative Z-stack confocal immunofluorescence image of NMO-rSkM cross-sections stained for DESMIN (red) and TUJ1 (green). Nuclei are counterstained with Hoechst (blue). Scale bar, 200 µm. **c.** Representative Z-stack confocal immunofluorescence image of NMO-rSkM cross-sections stained for SOX2 (green) and PAX6 (red). Nuclei are counterstained with Hoechst (blue). Scale bar, 10 µm. **d**. Representative Z-stack confocal immunofluorescence image of NMO-rSkM cross-sections stained HB9 (red). Nuclei are counterstained with Hoechst (blue). Scale bar, 10 µm. **e.** *HB9 and CHAT* gene expression in NMOs and NMO-rSKM. Data normalized to housekeeping *GAPDH* gene expression and shown as fold change over NMO. Data are shown as mean ± s.d. of 3 independent biological replicates. **f**. Representative Z-stack confocal immunofluorescence image of NMO-rSkM cross-sections stained for TUJ1 (red) and DESMIN (cyan). Nuclei are counterstained with Hoechst (grey). Scale bar, 50 µm. **g**. Representative Z-stack confocal immunofluorescence image of NMO-rSkM cross-sections stained for TUJ1 (red), S100B (cyan) and BTX (green). Nuclei are counterstained with Hoechst (grey). Scale bar, 50 µm. **h**. Representative stereomicroscope images of bundles of myofibers in NMO-rSkMs loaded with Fluo-4 before (upper panel) and after (lower panel) glutamate (Glu) stimulation. Scale bar, 500 µm. **i-j**. Representative quantification of normalized fluorescence intensity variation registered during live imaging analysis of NMO-rSkMs or NMO-rSkMs treated for 12 hours with BTX, and stimulated with Glu (f) or ACh (g). Each curve represents an individual ROI. 5 ROIs were analyzed per each biological sample. 3 independent replicates were analyzed for untreated NMO-rSkMs (light blue lines). 2 independent replicates were analyzed for NMO-rSkMs treated overnight with BTX (crimson lines). The dotted lines correspond to the baseline equal to 0.

**
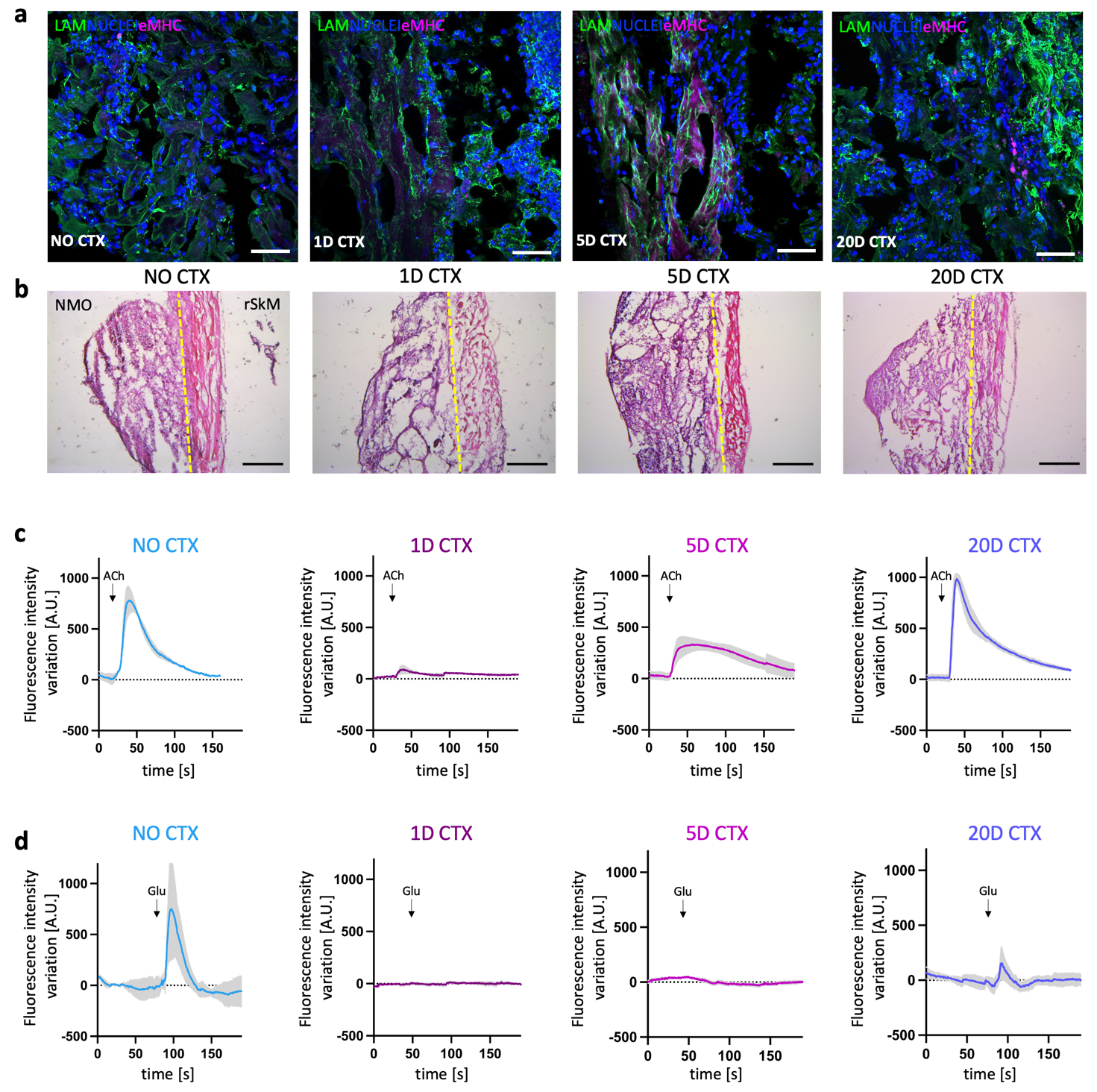
**

**Supplementary Figure 8**. **NMO-rSkM** **assembloid** **characterization after cardiotoxin (CTX) damage. a**. Representative Z-stack confocal immunofluorescence image of NMO-rSkM cross-sections stained for LAMININ (green) and embryonic MHC (magenta). Nuclei are counterstained with Hoechst (blue). Scale bars, 50 μm. **b**. Hematoxylin and eosin on D35 NMO-rSkM samples not treated with cardiotoxin (no CTX) or 1 day, 5 days and 20 days after CTX treatment. Scale bars, 500 µm (upper panel), 200 µm (lower panel). **c**. Representative quantification of mean normalized fluorescence intensity variation registered during contraction of NMO-rSkMs at day 35 of co-culture not treated with cardiotoxin (no CTX) or 1 day, 5 days and 20 days after CTX treatment when stimulated with ACh. For each curve, data are shown as mean ± SEM of 3 independent replicates. **d**. Representative quantification of mean normalized fluorescence intensity variation registered during contraction of NMO-rSkMs at day 35 of co-culture not treated with cardiotoxin (no CTX) or 1 day, 5 days and 20 days after CTX treatment when stimulated with Glu. For each curve, data are shown as mean ± SEM of 3 independent replicates.

**Supplementary Table 1. Statistical analyses of Figure 2b.**

| **Mann Whitney test** | **Significant** | **Summary** | **P Value** |
| --- | --- | --- | --- |
| rSkM vs NMO-dSkM | Yes | **** | <0.0001 |
| NMO-dSkM vs NMO-rSkM | Yes | **** | <0.0001 |
| rSkM vs NMO-rSkM | Yes | **** | <0.0001 |

**Supplementary Table 2. Statistical analyses of Figure 2c.**

| **Mann Whitney test** | **Significant** | **Summary** | **P Value** |
| --- | --- | --- | --- |
| rSkM vs NMO-dSkM | Yes | **** | <0.0001 |
| NMO-dSkM vs NMO-rSkM | Yes | **** | <0.0001 |
| rSkM vs NMO-rSkM | Yes | **** | <0.0001 |

**Supplementary Table 3. Statistical analyses of Figure 2e.**

| **Mann Whitney test** | **Significant** | **Summary** | **P Value** |
| --- | --- | --- | --- |
| rSkM vs NMO-dSkM | No | ns | 0.1000 |
| NMO-dSkM vs NMO-rSkM | No | ns | 0.0952 |
| rSkM vs NMO-rSkM | Yes | * | 0.0238 |

**Supplementary Table 4. Statistical analyses of Figure 2f.**

| **Mann Whitney test** | **Significant** | **Summary** | **P Value** |
| --- | --- | --- | --- |
| rSkM vs NMO-dSkM | No | ns | 0.1000 |
| NMO-dSkM vs NMO-rSkM | No | ns | 0.2619 |
| rSkM vs NMO-rSkM | Yes | * | 0.0238 |

**Supplementary Table 5. Statistical analyses of Figure 2o.**

| **Mann Whitney test** | **Significant** | **Summary** | **P Value** |
| --- | --- | --- | --- |
| rSkM vs NMO-dSkM | No | ns | 0.2496 |
| NMO-dSkM vs NMO-rSkM | Yes | ** | 0.0066 |
| rSkM vs NMO-rSkM | Yes | *** | 0.0001 |

**Supplementary Table 6. Statistical analysis of Figure 3n.**

| **Mann Whitney test** | **Significant** | **Summary** | **P Value** |
| --- | --- | --- | --- |
| rSkM vs NMO-rSkM | Yes | **** | <0.0001 |
| NMO-rSkM vs NMO-rSkM 12h BTX | Yes | **** | <0.0001 |
| rSkM vs NMO-rSkM 12h BTX | Yes | **** | <0.0001 |

**Supplementary Table 7. Statistical analysis of Figure 3p.**

| **Mann Whitney test** | **Significant** | **Summary** | **P Value** |
| --- | --- | --- | --- |
| rSkM vs NMO-rSkM | No | ns | 0.0713 |
| NMO-rSkM vs NMO-rSkM 12h BTX | Yes | **** | <0.0001 |
| rSkM vs NMO-rSkM 12h BTX | Yes | **** | <0.0001 |

**Supplementary Table 8. Statistical analysis of Figure 4e.**

| **Mann Whitney test** | **Significant** | **Summary** | **P Value** |
| --- | --- | --- | --- |
| NO CTX vs 1D CTX | Yes | **** | <0.0001 |
| NO CTX vs 5D CTX | Yes | **** | <0.0001 |
| NO CTX vs 20D CTX | No | ns | 0.1305 |
| 1D CTX vs 5D CTX | No | ns | 0.0833 |
| 1D CTX vs 20D CTX | Yes | **** | <0.0001 |
| 5D CTX vs 20D CTX | Yes | **** | <0.0001 |

**Supplementary Table 9. Statistical analysis of Figure 4f.**

| **Mann Whitney test** | **Significant** | **Summary** | **P Value** |
| --- | --- | --- | --- |
| NO CTX vs 1D CTX | Yes | **** | <0.0001 |
| NO CTX vs 5D CTX | Yes | **** | <0.0001 |
| NO CTX vs 20D CTX | Yes | **** | <0.0001 |
| 1D CTX vs 5D CTX | Yes | **** | <0.0001 |
| 1D CTX vs 20D CTX | Yes | **** | <0.0001 |
| 5D CTX vs 20D CTX | Yes | **** | <0.0001 |

**Supplementary Table 10. Statistical analysis of Figure 4g.**

| **Mann Whitney test** | **Significant** | **Summary** | **P Value** |
| --- | --- | --- | --- |
| NO CTX vs 1D CTX | Yes | **** | <0.0001 |
| NO CTX vs 5D CTX | Yes | **** | <0.0001 |
| NO CTX vs 20D CTX | Yes | **** | <0.0001 |
| 1D CTX vs 5D CTX | Yes | **** | <0.0001 |
| 1D CTX vs 20D CTX | Yes | **** | <0.0001 |
| 5D CTX vs 20D CTX | Yes | **** | <0.0001 |

**Supplementary Table 11. Statistical analysis of Figure 4i.**

| **Mann Whitney test** | **Significant** | **Summary** | **P Value** |
| --- | --- | --- | --- |
| NO CTX vs 1D CTX | Yes | **** | <0.0001 |
| NO CTX vs 5D CTX | Yes | **** | <0.0001 |
| NO CTX vs 20D CTX | Yes | **** | <0.0001 |
| 1D CTX vs 5D CTX | Yes | **** | <0.0001 |
| 1D CTX vs 20D CTX | Yes | * | 0.0218 |
| 5D CTX vs 20D CTX | Yes | **** | <0.0001 |

**Supplementary Table 12. Statistical analysis of Figure 4j.**

| **Mann Whitney test** | **Significant** | **Summary** | **P Value** |
| --- | --- | --- | --- |
| NO CTX vs 1D CTX | Yes | **** | <0.0001 |
| NO CTX vs 5D CTX | Yes | **** | <0.0001 |
| NO CTX vs 20D CTX | No | ns | 0.4801 |
| 1D CTX vs 5D CTX | Yes | **** | <0.0001 |
| 1D CTX vs 20D CTX | Yes | ** | 0.0013 |
| 5D CTX vs 20D CTX | Yes | **** | <0.0001 |

**Supplementary Table 13. Statistical analysis of Figure 4k.**

| **Mann Whitney test** | **Significant** | **Summary** | **P Value** |
| --- | --- | --- | --- |
| NO CTX vs 1D CTX | Yes | **** | <0.0001 |
| NO CTX vs 5D CTX | Yes | ** | 0.0020 |
| NO CTX vs 20D CTX | No | ns | 0.7478 |
| 1D CTX vs 5D CTX | Yes | ** | 0.0030 |
| 1D CTX vs 20D CTX | Yes | **** | <0.0001 |
| 5D CTX vs 20D CTX | Yes | ** | 0.0015 |

**Supplementary Table 14. Statistical analysis of Figure 5b.**

| **Tukey's multiple comparisons test** | **Significant** | **Summary** | **P Value** |
| --- | --- | --- | --- |
| NO CTX vs. 1D | Yes | **** | <0.0001 |
| NO CTX vs. 5D | Yes | **** | <0.0001 |
| NO CTX vs. 20D | Yes | **** | <0.0001 |
| 1D vs. 5D | Yes | **** | <0.0001 |
| 1D vs. 20D | Yes | **** | <0.0001 |
| 5D vs. 20D | Yes | **** | <0.0001 |

**Supplementary Table 15. Statistical analysis of Figure 5d.**

| **Mann Whitney test** | **Significant** | **Summary** | **P Value** |
| --- | --- | --- | --- |
| NO CTX vs 1D CTX | Yes | **** | <0.0001 |
| NO CTX vs 5D CTX | Yes | ** | 0.0012 |
| NO CTX vs 20D CTX | No | ns | 0.1160 |
| 1D CTX vs 5D CTX | Yes | * | 0.0113 |
| 1D CTX vs 20D CTX | Yes | **** | <0.0001 |
| 5D CTX vs 20D CTX | Yes | **** | <0.0001 |

**Supplementary Table 16. Statistical analysis of Figure 5g.**

| **Tukey's multiple comparisons test** | **Significant** | **Summary** | **P Value** |
| --- | --- | --- | --- |
| NO CTX vs. 1D | Yes | **** | <0.0001 |
| NO CTX vs. 5D | Yes | **** | <0.0001 |
| NO CTX vs. 20D | Yes | * | 0.0303 |
| 1D vs. 5D | Yes | **** | <0.0001 |
| 1D vs. 20D | Yes | **** | <0.0001 |
| 5D vs. 20D | Yes | **** | <0.0001 |

**Supplementary Table 17. Statistical analysis of Figure 5i.**

| **Mann Whitney test** | **Significant** | **Summary** | **P Value** |
| --- | --- | --- | --- |
| NO CTX vs 1D CTX | Yes | **** | <0.0001 |
| NO CTX vs 5D CTX | Yes | **** | <0.0001 |
| NO CTX vs 20D CTX | Yes | **** | <0.0001 |
| 1D CTX vs 5D CTX | Yes | **** | <0.0001 |
| 1D CTX vs 20D CTX | Yes | **** | <0.0001 |
| 5D CTX vs 20D CTX | Yes | *** | 0.0002 |

**Supplementary Table 18.** List of primary antibodies used in this study.

| **Antibody** | **Host** | **Dilution** | **Company** |
| --- | --- | --- | --- |
| Adult Myosin Heavy Chain (MYHC) | Mouse | 1:25 | DSHB (MF-20) |
| Adult Myosin Heavy Chain (MYHC) | Mouse | 1:200 | R&D Systems (MAB4470) |
| DESMIN | Rabbit | 1:300 | Abcam (ab15200) |
| DESMIN | Mouse | 1:75 | Agilent DAKO (M0760) |
| Green Fluorescent Protein (GFP) | Chicken | 1:300 | Millipore (06-896) |
| Homeobox transcription factor (HB9) | Mouse | 1:25 | DSHB (81.5C10) |
| Ki67 | Rabbit | 1:200 | Abcam (Ab16667) |
| Laminin (LAM) | Rabbit | 1:300 | Sigma-Aldrich (L9393) |
| Laminin (LAM) | Rat | 1:100 | Sigma-Aldrich (L0663) |
| MYOD | Mouse | 1:200 | Santa Cruz Biotechnology (sc-377460) |
| Myogenin (MYOG) | Rabbit | 1:25 | Santa Cruz Biotechnology (sc-576) |
| Myogenin (MYOG) | Rabbit | 1:200 | Abcam (124800) |
| Myosin Heavy Chain 1 (SlowMHC) | Mouse | 1:50 | DSHB (BA-D5) |
| Myosin Heavy Chain 2 (FastMHC) | Mouse | 1:50 | DSHB (sc-71) |
| Myosin Heavy Chain 3 (eMHC) | Mouse | 1:25 | DSHB (F1.652) |
| Neurofilament | Rabbit | 1:200 | Sigma-Aldrich (N4142) |
| Neuron-specific class III beta-tubulin (TUJ1) | Mouse | 1:5000 | Biolegend (MMS-435P) |
| Paired box protein (PAX6) | Rabbit | 1:100 | Biolegend (901301) |
| Paired box protein (PAX7) | Mouse | 1:25 | DSHB (PAX7s) |
| Paired box protein (PAX7) | Mouse | 1:50 | Santa Cruz Biotechnology (sc-81648) |
| S-100 protein beta chain (S100β) | Rabbit | 1:100 | Abcam (ab52642) |
| SRY – Box transcription factor 2 (SOX2) | Goat | 1:200 | R&D Systems (AF2018) |
| Sarcomeric Alpha Actinin | Mouse | 1:200 | Abcam (ab9465) |
| TE7 | Mouse | 1:100 | Millipore (CBL271) |
| Titin | Mouse | 1:50 | Santa Cruz Biotechnology (sc-271946) |

**Supplementary Table 19.** List of secondary antibodies and fluorescent dyes used in this study.

| **Antibody** | **Host** | **Dilution** | **Company** |
| --- | --- | --- | --- |
| anti-rabbit 488 | Donkey | 1:500 | Invitrogen (A21206) |
| anti-rabbit 594 | Donkey | 1:500 | Invitrogen (A21207) |
| anti-rabbit 647 | Donkey | 1:500 | Life Tech (A31573) |
| anti-mouse 488 | Donkey | 1:500 | Invitrogen (A21202) |
| anti-mouse 594 | Donkey | 1:500 | Invitrogen (A21203) |
| anti-mouse 647 | Donkey | 1:500 | Life Tech (A31571) |
| anti-goat 488 | Donkey | 1:500 | Life Technologies (A11055) |
| anti-chicken 488 | Goat | 1:500 | Abcam (Ab150169) |
| Anti-rat 488 | Goat | 1:500 | Invitrogen (A11006) |
| Phalloidin (F-Actin) 488 |  | 1:300 | Invitrogen (A12379) |
| Phalloidin (F-Actin) 647 |  | 1:300 | Invitrogen (A22287) |
| α-Bungarotoxin 555 |  | 1:1000 | Life Technologies (B35451) |
| α-Bungarotoxin 647 |  | 1:1000 | Invitrogen (B35450) |

**Supplementary Table 20. List of primers used in this study.** Oligonucleotide sequences specific for the different genes of interest were used to quantify gene expression. GAPDH and B2 microgobulin were used as housekeeping genes.

| **Gene** | **Primer probe** | **Sequence** | **References** |
| --- | --- | --- | --- |
| Acetylcholine Receptor Epsilon (ACHRE) | Forward  Reverse | GCCTGAGGATACTGTCACCATC  GTCCTTGCTGTAGTTGAGTCGG | OriGene  NM_000080 |
| Acetylcholine Receptor Gamma (ACHRG) | Forward  Reverse | CTGTCTTCCTCTTCCTTGTGGC  CGACAATGAGGATGGTCACCAC | OriGene  NM_005199 |
| B2 microglobulin (B2M) | Forward  Reverse | CAACTTCAATGTCGGATGGATG  GCTGTGCTCGCGCTACTCT | Trevisan C et al., 2019 |
| Choline acetyltransferase (CHAT) | Forward  Reverse | ACACTCCTGAGTGGTGCG  TTTTCCAGGATGGGCGTCTTG | Lee HJ et al., 2014 |
| GAPDH | Forward  Reverse | AAGGGCTCATGACCACAGTC  GGATGACCTTGCCCACAG | Tian et al., 2013 |
| LIM/homeodomain family of transcription factors (ISLET-1) | Forward  Reverse | TGCGCCAAGTGCAGCAT  AGCGGGCACGCATCAC | Lee HJ et al., 2014 |
| MN-determinant homeobox gene (HB9) | Forward  Reverse | CACCGAGACCCAGGTGAAGATTT  CCCTTCTGTTTCTCCGCTTCCT | Spitalieri P et al., 2018 |
| Myogenin (MyoG) | Forward  Reverse | CTGTCTTCCTCTTCCTTGTGGC  CGACAATGAGGATGGTCACCAC | Al Tanoury et al., development 2020 |
| Myosin Heavy Chain (MyHC,pan) | Forward  Reverse | CAGCCTGGAGCAGCTGTGCAT  ATGCCCATAGGCTTCTCGATGAGCTC | Trevisan C et al., 2019 |
| Myosin Heavy Chain 2A (MYHC 2A) | Forward  Reverse | AGGCTTCAAGATTTGGTAGA  TTCCTTTGCAACAGGGTAGA | Y. Liu et al., 2003 |
| Pax7 | Forward Reverse | ACCCCTGCCTAACCACATC  GCGGCAAAGAATCTTGGAGAC | Al Tanoury et al., development 2020 |

**Supplementary Video 1. Live imaging of rSkM, NMO-dSkM and NMO-rSkM showing contraction and calcium transients upon Acetylcholine administration.** Representative Fluo-4 calcium dye time-lapse stereomicroscope imaging of rSkM, NMO-dSkM and NMO-rSkM D35 representative samples, upon ACh administration. Acquisition frame rate: 5 fps. Shown frame rate: 100 fps. Scale bars, 1 mm.

**Supplementary Video 2. Live imaging showing NMO-rSkM contraction and calcium transients upon Glutamate administration, before and after BTX treatment.** Fluo-4 calcium dye time-lapse stereomicroscope imaging of D35 NMO-rSkM and D35 NMO-rSkM treated for 12h with BTX upon ACh administration. Representative samples. Acquisition frame rate: 5 fps. Shown frame rate: 40 fps. Scale bar, 1 mm.

**Supplementary Video 3. Live imaging showing NMO-rSkM contraction and calcium transients upon Acetylcholine administration, before and after BTX treatment.** Fluo-4 calcium dye time-lapse stereomicroscope imaging of D35 NMO-rSkM and D35 NMO-rSkM treated for 12h with BTX upon ACh administration. Representative samples. Acquisition frame rate: 5 fps. Shown frame rate: 40 fps. Scale bar, 1 mm.

**Supplementary Video 4. Live imaging showing NMO-rSkM contraction and calcium transients upon Acetylcholine administration, before and after CTX treatment.** Fluo-4 calcium dye time-lapse stereomicroscope imaging of D35 NMO-rSkM and D35 NMO-rSkM treated with CTX after 1 day (1D), 5 days (5D) and 20 days (20D) upon ACh administration. Representative samples. Acquisition frame rate: 5 fps. Shown frame rate: 80 fps. Scale bar, 1 mm.

**Supplementary Video 5. Live imaging showing NMO-rSkM contraction and calcium transients upon Glutamate administration, before and after CTX treatment.** Fluo-4 calcium dye time-lapse stereomicroscope imaging of D35 NMO-rSkM and D35 NMO-rSkM treated with CTX after 1 day (1D), 5 days (5D) and 20 days (20D) upon Glu administration. Representative samples. Acquisition frame rate: 5 fps. Shown frame rate: 80 fps. Scale bar, 1 mm.
